# High Caveolin-1 mRNA expression in triple-negative breast cancer is associated with an aggressive tumor microenvironment, chemoresistance, and poor clinical outcome

**DOI:** 10.1371/journal.pone.0305222

**Published:** 2024-07-03

**Authors:** Christopher Godina, Somayeh Khazaei, Mattias Belting, Johan Vallon-Christersson, Björn Nodin, Karin Jirström, Karolin Isaksson, Ana Bosch, Helena Jernström

**Affiliations:** 1 Department of Clinical Sciences Lund, Oncology, Lund University and Skåne University Hospital, Lund, Sweden; 2 Department of Hematology, Oncology and Radiation Physics, Skåne University Hospital, Sweden; 3 Department of Immunology, Genetics and Pathology, Science for Life Laboratory, Uppsala University, Uppsala, Sweden; 4 Department of Clinical Sciences Lund, Oncology and Therapeutic Pathology, Lund University, Lund, Sweden; 5 Department of Clinical Sciences Lund, Surgery, Lund University and Kristianstad Hospital, Kristianstad, Sweden; The University of British Columbia Life Sciences Institute, CANADA

## Abstract

**Background:**

Currently, there are few treatment-predictive and prognostic biomarkers in triple-negative breast cancer (TNBC). Caveolin-1 (CAV1) is linked to chemoresistance and several important processes involved in tumor progression and metastasis, such as epithelial-mesenchymal transition (EMT). Herein, we report that high *CAV1* gene expression is an independent factor of poor prognosis in TNBC.

**Methods:**

*CAV1* gene expression was compared across different molecular features (e.g., PAM50 subtypes). *CAV1* expression was assessed in relation to clinical outcomes using Cox regression adjusted for clinicopathological predictors. Differential gene expression and gene set enrichment analyses were applied to compare high- and low-expressing *CAV1* tumors. Tumor microenvironment composition of high- and low-expressing *CAV1* tumors was estimated using ECOTYPER. Tumor tissue microarrays were used to evaluate CAV1 protein levels in stromal and malignant cells.

**Results:**

In the SCAN-B (n = 525) and GSE31519 (n = 327) cohorts, patients with *CAV1-*high tumors had an increased incidence of early recurrence adjusted HR 1.78 (95% CI 1.12–2.81) and 2.20 (95% CI 1.39–3.47), respectively. In further analysis, high *CAV1* gene expression was associated with a molecular profile indicating altered metabolism, neovascularization, chemoresistance, EMT, suppressed immune response, and active tumor microenvironment. Protein levels of CAV1 in malignant and stromal cells were not correlated with *CAV1* gene expression.

**Conclusion:**

*CAV1* gene expression in TNBC is a biomarker that merits further investigation in clinical trials and as a therapeutic target.

## Introduction

Triple-negative breast cancer (TNBC), characterized by the absence of human epidermal growth factor receptor 2 (HER2) overexpression as well as estrogen receptor (ER) and progesterone receptor (PR) negativity [1–3], accounts for approximately 10% of incident breast cancers and has the poorest prognosis among breast cancer subtypes [1–3]. TNBC is a subtype with few targeted treatments and is also biologically aggressive [1–3]. Nonetheless, TNBC is a remarkably heterogeneous disease [1–3]. Efforts have been made to characterize specific molecular subtypes of TNBC, the most well-known being the Lehman TNBC subtypes [4, 5]. However, the clinical implications of molecular profiling are still unclear [1–3]. In recent years, new treatments, such as immune-checkpoint inhibitors [6] and poly (ADP-ribose) polymerase (PARP) inhibitors [7], have been introduced. In the advanced setting, a new antibody-drug conjugate Sacituzumab-govitecan (targeting TROP2), has also been added [1–3]. Still, chemotherapy remains the primary systemic treatment for TNBC [1–3]. Taxanes and anthracyclines are effective treatments for TNBC, but a substantial proportion of patients relapse early [1–3, 8]. TNBC is a complex disease for which there is a need to find specific biomarkers to further stratify patients and help guide treatment decisions.

Emerging evidence suggests a role for Caveolin-1 (CAV1) in cytotoxic drug resistance [9, 10]. Tumors with higher *CAV1* expression have been linked to taxane resistance in both preclinical and clinical studies [9, 10]. Recently, a translational study within the GeparSepto trial reported that *CAV1* expression predicted a worse response to paclitaxel and worse clinical outcome in these patients [11]. These findings merit further investigation into CAV1 as a biomarker and potentially as a therapeutic target in TNBC. CAV1 constitutes the principal component of caveolae, which function as a hub for cell signaling and membrane transport of nutrients and substances, including drugs [12, 13]. As a master regulator of signal transduction, CAV1 plays an essential role in tumor-stroma interactions, hypoxia response, cellular metabolism, inflammation, and epithelial-mesenchymal transition (EMT) [12–14], which are critical drivers of tumor progression and metastasis.

The role of the tumor microenvironment (TME) is increasingly recognized as essential for tumor survival, growth, and metastasis [15, 16]. This has led to a more holistic view of the TNBC as a coordinated ecosystem, integrating the malignant cells and TME [1–3]. Therefore, the TME, mainly comprised of stromal and immune cells, may harbor relevant biomarkers and potential treatment targets for TNBC. Notably, CAV1 protein expression in stromal cells has been reported as a potential prognostic biomarker in breast cancer [13, 17–19]. To date, no large-scale studies have evaluated CAV1 as a biomarker in TNBC, nor has CAV1 been characterized thoroughly in the context of the TNBC TME.

In this study, we investigated both gene and protein expression of CAV1 in TNBC in several large cohorts, focusing on potential associations between CAV1 and molecular features, tumor microenvironment composition, and clinical outcome.

## Methods

First, *CAV1* gene expression was investigated in the Gene expression-based Outcome for Breast cancer Online (GOBO) platform. GOBO includes 1881 breast tumors with available follow-up for survival analysis that can be stratified by molecular subtype [20]. The GOBO platform is a versatile and user-friendly online tool designed for conducting various analyses on an 1881-sample breast tumor dataset generated using Affymetrix U133A microarrays [20]. GOBO functionalities include rapid evaluation of gene expression levels in different subgroups of breast tumors and examining the association between gene expression levels of individual genes and outcomes; details on the different analyses are described elsewhere [20].

Second, the Swedish Cancerome Analysis Network–Breast (SCAN-B: ClinicalTrials.gov ID NCT02306096) study was used. The SCAN-B study is a population-based cohort that prospectively includes breast cancer patients diagnosed and treated at nine Swedish hospitals [21, 22]. All newly diagnosed breast cancer patients are invited to participate [22].

Gene expression profiling of fresh tumor samples and core needle biopsies (in case of neoadjuvant treatment) was performed using RNA-seq according to custom SCAN-B workflow, as previously described [21–23]. The samples were obtained in conjunction with routine clinical sampling at the time of surgery [21, 22]. Gene expression levels were expressed in fragments per kilobase of exon per million mapped reads (FPKM) in an expression matrix for SCAN-B [23]. Clinicopathological data, treatment information, and follow-up were collected from the Swedish National Quality Registry for Breast Cancer [21–23].

**Fig 1:**
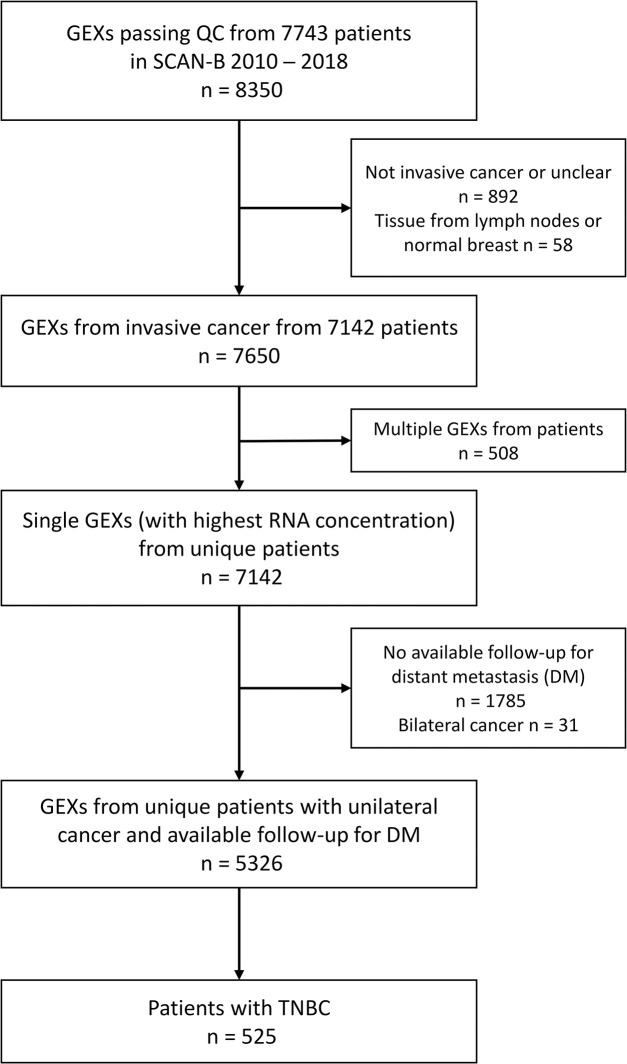
Flowchart of included and excluded patients in SCAN-B GEX.

Curated RNA-seq and clinicopathological data were accessed from the Supplementary Information and Data from Staaf *et al*. [23] for 7743 patients enrolled in SCAN-B from September 1, 2010, to May 31, 2018, and who were followed until 2021 [23]. To all FPKM data, an offset of +0.1 was added, and thereafter, the data was log2 transformed. Patients with gene expression profiles (GEXs) only from noninvasive cancer, lymph nodes, or bilateral cancer or who had no available follow-up for distant metastasis were excluded. In cases where multiple GEXs from a single tumor passed quality control, the GEX profile with the highest RNA concentration measured by NanoDrop spectrophotometry was chosen [23], leaving one GEX per patient for analysis. This procedure left a total of 5326 patients, of whom 525 had TNBC. According to Swedish National Guidelines, tumors are considered triple-negative if ER and PR staining is positive in less than 10% of tumor cells and HER2 is either 0/1+ by IHC assessment or non-amplified by in situ hybridization (ISH) assessment if the IHC score is 2+. These 525 TNBCs were used for further analysis and are hereafter referred to as SCAN-B GEX, Fig 1.

A subcohort of TNBC patients from SCAN-B was used to investigate CAV1 protein levels in different spatial localizations of TNBC [24]. The cohort consisted of patients from a Region Skåne hospital diagnosed with TNBC between 2010 and 2015 [24]. Tumor tissue microarrays (TMAs) were constructed for these patients from tumor tissue obtained at the time of surgery. Exclusion criteria for this cohort were inconsistency in TNBC status after clinical chart review, insufficient tumor material, failed RNA-seq quality filters, or formalin-fixed paraffin-embedded (FFPE) tissue not available for analysis in the TMA [24].

The TMAs were constructed as previously described [24], with duplicate cores of 1.0 mm from each TNBC. IHC for CAV1 was performed using the same protocol and antibody as previously described [25]. In brief, TMA slides (4 μm) were deparaffinized and pretreated using the PT Link system (Agilent Technologies, Santa Clara, CA, USA). Sections were then stained for CAV1 with a primary rabbit polyclonal anti-CAV1 antibody (1:1,000; ab2910, Abcam) using the Autostainer Plus with the EnVision FLEX high-pH kit, according to the manufacturer’s instructions (Agilent Technologies). Immunohistochemical staining was done on March 30, 2022. CAV1 was scored in the cytoplasm of both malignant and stromal cells according to the intensity of staining across the two tumor cores. If at least 20% of the cells were stained, the intensity was denoted as 1+ (weak staining), 2+ (moderate), or 3+ (strong) as per previous protocols [25]. If less than 20% of the tumor cells were stained, the staining was denoted as 0 (negative) as per previous protocols [17, 25]. The malignant and stromal cells were distinguished by morphological assessment per previous studies [17–19]. Scoring was performed by two independent readers (C. Godina and S. Khazaei), and in case of disagreement, a more experienced evaluator (B. Nodin) was consulted, and consensus was reached. All evaluators were blinded to data pertaining to the tumor samples. The protein levels of CAV1 in malignant and stromal cells were dichotomized as “strong” (1) vs.”negative to moderate” (0). The CAV1 categories in malignant and stromal cells, respectively, were combined to create a joint CAV1 status with four categories: malignant/stromal cells 0/0, 0/1, 1/0, and 1/1. CAV1 status could be evaluated in 231 of 242 tumors, which were included in the analysis, and this subcohort is hereafter referred to as SCAN-B TMA, S1 Fig in S1 File.

### Validation cohorts

Two additional cohorts, Molecular Taxonomy of Breast Cancer International Consortium (METABRIC) and GSE31519, were used to validate the findings in GOBO and SCAN-B. METABRIC consists of clinically annotated primary fresh-frozen breast cancer specimens from patients diagnosed with non-metastatic breast cancer between 1977 and 2005 in the UK and Canada [26]. Gene-expression data from microarrays is available for a subset of 1980 patients, known as the METABRIC molecular dataset [26–28]. Further details on clinicopathological data, sample handling, gene expression profiling, and quality control are described elsewhere [26, 28]. The METABRIC molecular dataset was accessed from https://www.cbioportal.org/study/summary?id=brca_metabric and corresponding clinical data from Rueda *et al*. [27]. Out of the 1980 patients, 320 had TNBC and were included in the analysis. The GSE31519 cohort consisted of pooled datasets from a single platform (Affymetrix U133A and U133 Plus 2.0 chips) and included only TNBC (n = 579) from 28 different datasets [29]. Follow-up was available for 327 TNBCs. Details on pooling, quality control, and analysis pipeline are available elsewhere [29]. If multiple probes mapped to the same gene, the average expression of the probes was used to represent the gene expression for the gene in question. GSE31519 data were downloaded from the GEO database (http://www.ncbi.nlm.nih.gov/geo/), accession identification number GSE31519.

### Gene expression analyses

*CAV1* expression data for all three cohorts was divided into tertiles, with tertile three defined as *CAV1-*high and tertiles one and two combined into *CAV1-*low, based on the GOBO results.

In SCAN-B, both PAM50 subtypes and ROR categories were assigned with single sample predictors and obtained from Staaf et al. [23]. For METABRIC and GSE31519, PAM50 subtypes were assigned using the *genefu* package [30] using nearest centroid correlation [31]. The PAM50 ROR score was calculated based on centroid correlations, tumor size, and proliferation score according to the ROR equation with nodal status-dependent cut-offs to assign ROR categories, as previously described [32–34]. All tumors were assigned a PAM50 subtype, but the ROR category was missing for tumors with missing data on tumor size and/or nodal status.

For TNBC type classification in all three cohorts [4], gene expression data of TNBCs were extracted and uploaded as a separate dataset into the web-based classifier [4]. For some tumors, the web-based application called the tumor as not being ER-negative. These tumors were removed from the TNBC datasets (inferring missing values), and the remaining tumors were again uploaded to the web-based application for subtyping. Further, eight gene expression modules representing different biological functions in breast cancer were calculated for all three cohorts as previously described [35].

Differential gene expression (DGE) analysis was performed using the Limma-Voom package [36] to find differentially expressed genes (DEGs) between *CAV1*-high and *CAV1-*low tumors in SCAN-B GEX. The criteria used to define DEGs is a false discovery rate (FDR) of ≤ 0.05 and log2 fold change (log2FC) ≥ 1.5 for up-regulated genes and log2FC ≤ −1.5 for down-regulated genes. To find concordant gene sets that differed between *CAV1*-high and *CAV1*-low, gene set enrichment analysis (GSEA) was performed in clusterProfiler [37]. Gene sets were grouped according to Gene Ontology (GO) and Hallmark Signature annotations [38, 39].

Furthermore, *CAV1* expression was profiled in the single-cell atlas of human breast cancers [40] using the Broad Institute Single Cell portal to investigate in which cell (sub)types *CAV1* was expressed. *In silico* profiling of different cell states and carcinoma ecosystems (including estimates of relative abundance) was derived from bulk RNA-seq data from SCAN-B GEX using a deconvolution-based method, ECOTYPER (with standard parameters) [41]. ECOTYPER applies a machine-learning framework for large-scale identification of cell states and cellular ecosystems from bulk gene expression data [41]. The average abundance of each cell state for said cell type was used to infer the relative abundance of cell types.

### Statistical analysis

Differences in log2 transformed *CAV1* expression depending on PAM50 subtype and TNBC subtype were evaluated using analysis of variance (ANOVA) and visualized using violin and box plots. The unpaired t-test was used to evaluate differences in the relative abundance of fibroblast and endothelial cells between *CAV1-*high and low tumors. Correlations between log2 transformed *CAV1* expression and the following variables: ROR category, the eight gene modules, fibroblast states, endothelial states, and carcinoma ecotypes (CE) were assessed using Pearson’s correlation (*r*). The correlations were visualized with bar plots. The dominant carcinoma ecotype was compared between *CAV1-*high and *CAV1-*low tumors using the Chi-square test.

For survival analyses, the R packages survival and survminer were used. The endpoints used were recurrence-free interval (RFI), distant metastasis-free interval (DMFI), and overall survival (OS) for both SCAN-B and METABRIC, as previously described [23, 26, 27]. The primary endpoint was DMFI. Breast cancer-specific survival (BCSS) was used as an additional endpoint for METABRIC [26, 27]. For GSE31519, event-free survival (EFS) was the only available endpoint, and the end of follow-up was set at 10 years [29].

The Kaplan-Meier estimator and Log-rank test were used for univariable survival analyses. Crude and adjusted hazard ratios (HRs) with 95% confidence intervals (CI) were obtained from Cox proportional hazards models. The multivariable models were *a priori* adjusted for age (binned in five-year intervals for SCAN-B or continuous for METABRIC and GSE31519), tumor characteristics, axillary lymph node status (pN1/2/3), tumor size (pT2/3/4), grade (III vs. I or II), PAM50 ROR category (High vs Low/Intermediate), and (neo)adjuvant chemotherapy (yes vs. no). Schoenfeld’s residuals were used to graphically examine the proportional hazard assumption for the *CAV1* (dichotomous) classification of tumors in the adjusted models for all cohorts. The Akaike information criterion (AIC) was used to compare standard clinical models to clinical models, including *CAV1* expression (dichotomous) with or without PAM50 ROR score using the AICcmodavg package.

R version 4.2.2 was used for all statistical analyses. All *P*-values were two-tailed, and *P* -values should be interpreted without reference to cut-offs for significance with or without FDR adjustment. This study followed the Reporting Recommendations for Tumor Marker Prognostic Studies (REMARK) criteria [42].

### Ethics statement

Ethical approvals for the cohorts studied were obtained in relation to the primary projects and publications [21–23, 26–29]. All participants signed written informed consent. The TMA part of the study has received ethical approval (Dnr2009/658, Dnr2015/277, and Dnr2019/01252) from the Lund University Ethics Committee. No other separate approval was obtained for this specific study since it is otherwise based on previously published data. The study was conducted in accordance with the ethical principles of the Declaration of Helsinki.

## Results

### GOBO: High *CAV1* expression was associated with worse prognosis in ER-negative and basal tumors

**Fig 2:**
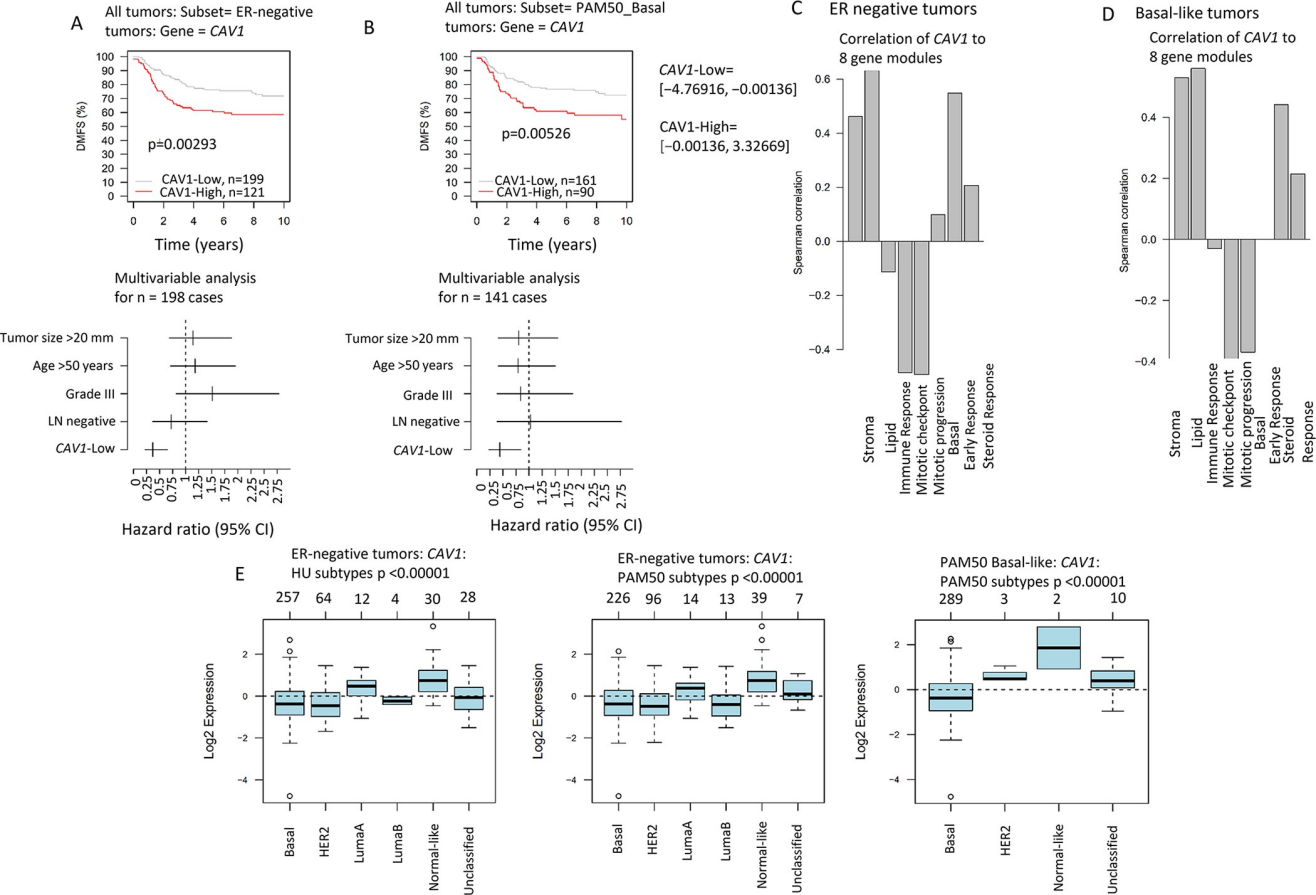
*CAV1* expression in GOBO. Kaplan-Meier estimates of *CAV1* expression (dichotomous) in and corresponding forest plots of mutually adjusted hazard ratios (95% confidence intervals) in (**A**) patients with ER negative breast cancer. Kaplan-Meier estimates of *CAV1* expression (dichotomous) in and corresponding forest plots of mutually adjusted hazard ratios (95% confidence intervals) in (**B**) patients with Basal-like breast cancer. Pearson correlations of *CAV1* gene expression (continuous) and the eight gene modules (stroma, lipid, immune response, mitotic checkpoint, mitotic progression, basal, early response, steroid response) in (**C**) patients with ER negative breast cancer. Pearson correlations of *CAV1* gene expression (continuous) and the eight gene modules (stroma, lipid, immune response, mitotic checkpoint, mitotic progression, basal, early response, steroid response) in (**D**) patients with basal-like breast cancer. The number of patients in each group at diagnosis is indicated as *n*. *CAV1* expression by intrinsic subtypes using PAM50 and Hu *et al*. [65] classifications in (**E**) the GOBO dataset.

Investigation of the GOBO database revealed that patients with ER-negative tumors with high *CAV1* expression had shorter distant metastasis-free survival (DMFS) in univariable and multivariable analyses compared to low *CAV1* expression, Fig 2A and 2B. The difference in DMFS was especially apparent in the subset of tumors classified as basal, Fig 2A and 2B. This finding, together with previous published results [13, 17–19], implies that *CAV1* expression is a potential prognostic marker in TNBC. Further analyses in GOBO pertaining to molecular features revealed that *CAV1* expression was highest in normal-like and second highest in luminal A tumors, Fig 2C–2E. *CAV1* expression was strongly correlated with stromal and lipid modules while negatively correlated with both mitotic modules (checkpoint and proliferation), indicating low proliferation, Fig 2C–2E.

### SCAN-B, METABRIC, and GSE31519: *CAV1* mRNA expression in relation to molecular and clinicopathological factors

**Fig 3:**
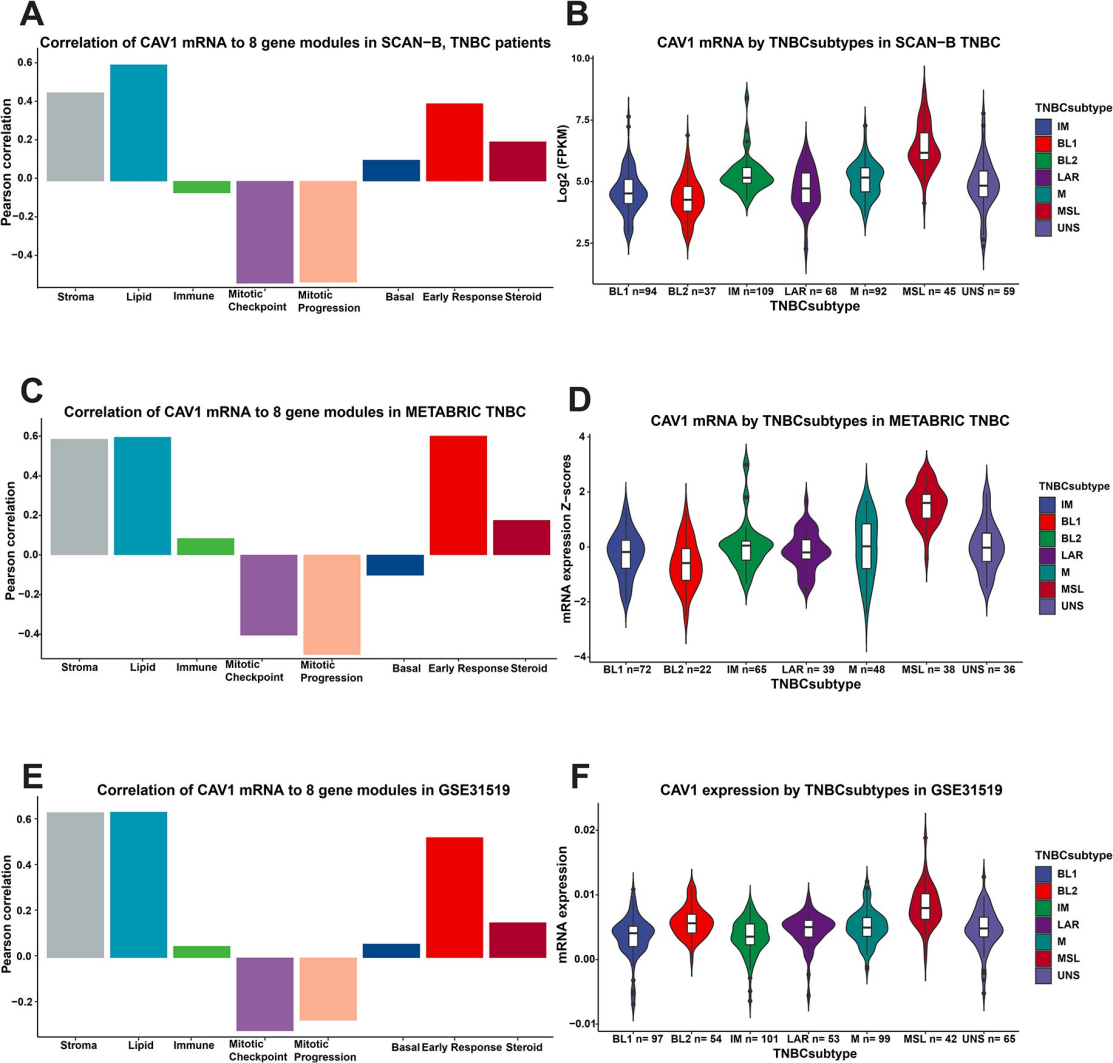
*CAV1* expression in relation to molecular features. *CAV1* expression (continuous) by TNBC molecular subtype in (**A**) SCAN-B GEX, (**B**) GSE31519, and (**C**) METABRIC. Pearson correlations of *CAV1* gene expression (continuous) and the eight gene modules (stroma, lipid, immune response, mitotic checkpoint, mitotic progression, basal, early response, steroid response) in (**D**) SCAN-B GEX, (**E**) GSE31519, and (**F**) METABRIC.

Similar to the findings in GOBO, *CAV1* expression in TNBC in all three cohorts was highest in the normal-like and second highest in the luminal A subtype (all *Ps* <0.001), S2A-S2C Fig in S1 File. There was also an inverse association with the ROR category in all three cohorts (all *Ps*<0.001), S2D-S2F Fig in S1 File. Likewise, the correlations between *CAV1* expression and the eight gene modules in TNBC were similar in all three cohorts and GOBO. There were strong positive correlations between *CAV1* expression and lipid and stroma modules and negative correlations with the mitotic checkpoint and progression modules, Fig 3A, 3C and 3E. The distribution of *CAV1* gene expression was similar across the TNBC subtypes in all cohorts, with the highest *CAV1* expression in the mesenchymal stem-like, followed by the Mesenchymal subtype (all *Ps*<0.001), Fig 3C, 3D and 3F. Descriptive statistics for clinicopathological factors in *CAV1-*high and *CAV1-*low tumors are presented in Table 1 for SCAN-B and S1 Table in S2 File for METABRIC and S2 Table in S2 File for GSE31519. Chemotherapy was markedly more common in the SCAN-B cohort compared with the other two cohorts.

**Table 1:** Descriptive statistics of *CAV1-*high and low tumors in SCAN-B GEX.

	SCAN-B, TNBC n = 525			
	All patients	Missing	*CAV1* mRNA expression n = 525	
			Low	High
	n = 525		n = 350	n = 175
	Number (%)		Number (%)	Number (%)
**Age at diagnosis, years**		0		
–40	62 (11.8)		47 (13.4)	15 (8.6)
41–50	77 (14.7)		51 (14.6)	26 (14.9)
51–60	114 (21.7)		83 (23.7)	31 (17.7)
61–70	128 (24.4)		73 (20.9)	55 (31.4)
71–80	87 (16.6)		55 (15.7)	32 (18.3)
81–	57 (10.9)		41 (11.7)	16 (9.1)
**Invasive tumor size**		32		
pT2/3/4	283 (57.4)		150 (45.0)	60 (37.5)
**Axillary lymph node involvement**		29		
pN1/2/3 (any)	167 (33.7)		109 (32.8)	58 (35.4)
**Main histological type**		3		
No special type (formerly ductal)	444 (85.1)		303 (87.1)	141 (81.0)
Lobular	12 (2.3)		2 (0.6)	10 (5.7)
Other or mixed	66 (12.6)		43 (12.4)	23 (13.2)
**Histological grade**		67		
I	7 (1.5)		4 (1.3)	3 (2.1)
II	65 (14.2)		29 (9.1)	36 (25.5)
III	386 (84.3)		284 (89.6)	102 (72.3)
**Systemic Treatment**				
Chemotherapy	393 (76.8)	13	260 (76.5%)	133 (77.3%)
**PAM50 Subtype**		0		
Luminal A	5 (1.0)		2 (0.6)	3 (1.7)
Luminal B	3 (0.6)		3 (0.9)	0 (0.0)
Normal-like	73 (13.9)		13 (3.7)	60 (34.3)
HER2 enriched	79 (15.0)		68 (19.4)	11 (6.3)
Basal	365 (69.5)		264 (75.4)	101 (57.7)
**PAM50 ROR**		45		
Low	52 (10.8)		12 (3.7)	40 (26.0)
Intermediate	32 (6.7)		11 (3.4)	21 (13.6)
High	396 (82.5)		303 (92.9)	93 (60.4)
**TNBC Subtype**		21		
BL1	94 (18.7)		83 (24.2)	11 (6.8)
BL2	37 (7.3)		22 (6.4)	15 (9.3)
IM	109 (21.6)		90 (26.2)	19 (11.8)
LAR	68 (13.5)		49 (14.3)	19 (11.8)
M	92 (18.3)		54 (15.7)	38 (23.6)
MSL	45 (8.9)		3 (0.9)	42 (26.1)
UNS	59 (11.7)		42 (12.2)	17 (10.6)

### SCAN-B: Relationship between CAV1 protein levels in different spatial localizations and clinicopathological, molecular factors, and *CAV1* gene expression

Strong CAV1 protein staining in malignant cells was associated with a higher histological grade but no axillary lymph node involvement (both *P*<0.007). In contrast, strong CAV1 protein staining in stromal cells was associated with lower histological grade but axillary lymph node involvement (both *P*<0.001), S3 Table in S2 File. Strong CAV1 protein staining in stromal cells was also associated with higher age at diagnosis (*P* = 0.025). With regards to PAM50 subtypes, strong CAV1 staining in malignant cells was positively associated with the basal subtype, while strong CAV1 staining in stromal cells was positively associated with the HER2 enriched subtype (*P*<0.001), S3A, S3B Fig in S1 File. Depending on spatial localization, strong CAV1 protein staining was associated with different TNBC subtypes. Strong CAV1 staining in malignant cells was positively associated with the mesenchymal and negatively associated with the immunomodulatory subtype (*P*<0.001). Strong CAV1 staining in stromal cells was positively associated with the luminal androgen (LAR) subtype (*P*<0.001), S3C, S3D Fig in S1 File. Neither CAV1 protein levels in malignant cells nor in stromal cells were correlated with *CAV1* gene expression in the tumors, and the combined CAV1 status was also not associated with *CAV1* gene expression, S3E-S3G Fig in S1 File.

### SCAN-B: DGE and GSEA analysis of *CAV1*-high vs. *CAV1*-low tumors

DGE analyses were performed in TNBCs, comparing *CAV1-*high versus *CAV1-*low tumors in SCAN-B to elucidate the potential biological role of *CAV1* in TNBC.

A total of 61 genes were found to be upregulated in *CAV1-*high versus *CAV1-*low tumors, and no genes were downregulated. Notably, higher expression of several genes coding for proteins involved in cellular lipid metabolism, e.g., *FABP4*, *IGF1*, *IGF2*, *LEP*, *TUSC5*, *CIDEA*, *HSPB6*, *LIPE*, *PLIN4*, *PLIN1 ADH1B*, and *ADH1C* were seen in *CAV1-*high tumors, supporting a potential association with altered tumor metabolism. Further, in *CAV1-*high tumors, genes related to endothelial cells, platelet activation, and vascular homeostasis (*ANGPT1*, *CTSG*, *LYVEL*, *CMA1*, *MMRN1*, *CCL14*, *TIMP4*, *SVEP1*, *PI16*, *ADAM33*, *VEGFD*, among others) were also upregulated, S4 Fig in S1 File and S4 Table in S3 File. Enriched gene sets in *CAV1-*high tumors included EMT, TGF-β signaling, adipogenesis, myogenesis, coagulation, angiogenesis, and hypoxia, among others, S4 Fig in S1 File and S5 Table in S3 File. In *CAV1-*low tumors, the G2M checkpoint, E2F targets, interferon alpha and beta response, MYC targets V1 and V2, UV damage response, and mTOR signaling hallmark gene sets, among others, were enriched, suggesting increased proliferation and immune response, S4 Fig in S1 File and S5 Table in S3 File. Similar patterns were seen regarding GO terms, S4 Fig in S1 File and S6 Table in S3 File.

### SCAN-B: Tumor microenvironment composition in relation to *CAV1* mRNA expression

The analysis of *CAV1* expression in the single-cell atlas of human breast cancers [40] revealed that *CAV1* is highly expressed in stromal cells in the order of endothelial cells, perivascular-like (PVL) cells, and CAFs while weakly expressed in malignant cells and barely expressed at all in immune cells, S5 Fig in S1 File. In the subpopulations of each stromal cell type, *CAV1* was most highly expressed in *CXCL12*^*+*^ endothelial cells, differentiated PVL, and myCAFs in each respective cell type (endothelial, PVL, CAF), S5 Fig in S1 File. The tumor microenvironment composition was estimated by ECOTYPER [41] to investigate whether the composition differed between *CAV1-*high and *CAV1*-low tumors in SCAN-B GEX. *CAV1-*high tumors had a higher relative abundance of endothelial and stromal cells compared to *CAV1-*low tumors (both *P*<0.001), S6 Fig in S1 File. Additionally, *CAV1-*high tumors were associated with the dominance of carcinoma ecotype (CE) 6 followed by CE 1 (*P*<0.001), S6 Fig in S1 File. This indicates that *CAV1-*high tumors have a microenvironment enriched for stromal cells while deficient in immune cells. CE 6 and 1 are characterized by POSTN^+^ fibroblasts and the putative binding of malignant cells’ ligands (*BST1 CYR61*, *GNA12*, *ICAM1*, *PTGS2*, and *TGFB1*, among others) to *CAV1* in endothelial cells. Further analysis of the different cell states revealed that *CAV1* expression was correlated to state 2 (S02) fibroblasts (CD34^+^ and SPARCL1^+^, CAF1; myofibroblast features) and S03 fibroblasts (COL10A1^+^ and POSTN^+^, CAF2; extra-cellular matrix remodeling features) [43]. High *CAV1* expression was also associated with S01 endothelial cells (CD36^+^, normal-enriched) and S02 endothelial cells (ANGPTL2^+^ and NID2^+^, neovascularization-associated) (all *P*<0.001), S6 Fig in S1 File. A negative correlation to S04 endothelial cells (ITGA3^+^ and IRF1^+^, unknown function) was also seen (*P*<0.001). These findings support a potential role in an active stromal component in TNBC that promotes vascularization and EMT as well as suppressing immune response.

### SCAN-B, METABRIC, and GSE31519: High *CAV1* mRNA expression confers inferior clinical outcomes

In the SCAN-B GEX cohort, the median follow-up for the 351 patients still at risk was 5.48 years (IQR 5.00–8.15). For the 146 patients still at risk in the SCAN-B TMA cohort, the median follow-up was 8.13 years (IQR 6.67–9.23). The follow-up was restricted to 10 years in METABRIC. All events after 10 years were censored to make METABRIC more comparable to SCAN-B and GSE31519. The median follow-up for the 173 patients still at risk in METABRIC was 10.0 years (IQR 9.82–10.0). For the GSE31519 dataset, the median follow-up was 7.79 years (IQR 5.69–10.00) for the 169 patients still at risk. The proportional hazards assumption was reasonably well fulfilled for the *CAV1* expression (dichotomous) for all endpoints in all cohorts.

Strong CAV1 protein staining in malignant and stromal cells of breast cancer tumors was not associated with clinical outcomes in either the univariable or multivariable survival analyses in the SCAN-B TMA cohort, S7 Fig in S1 File and S7 Table in S2 File.

**Fig 4:**
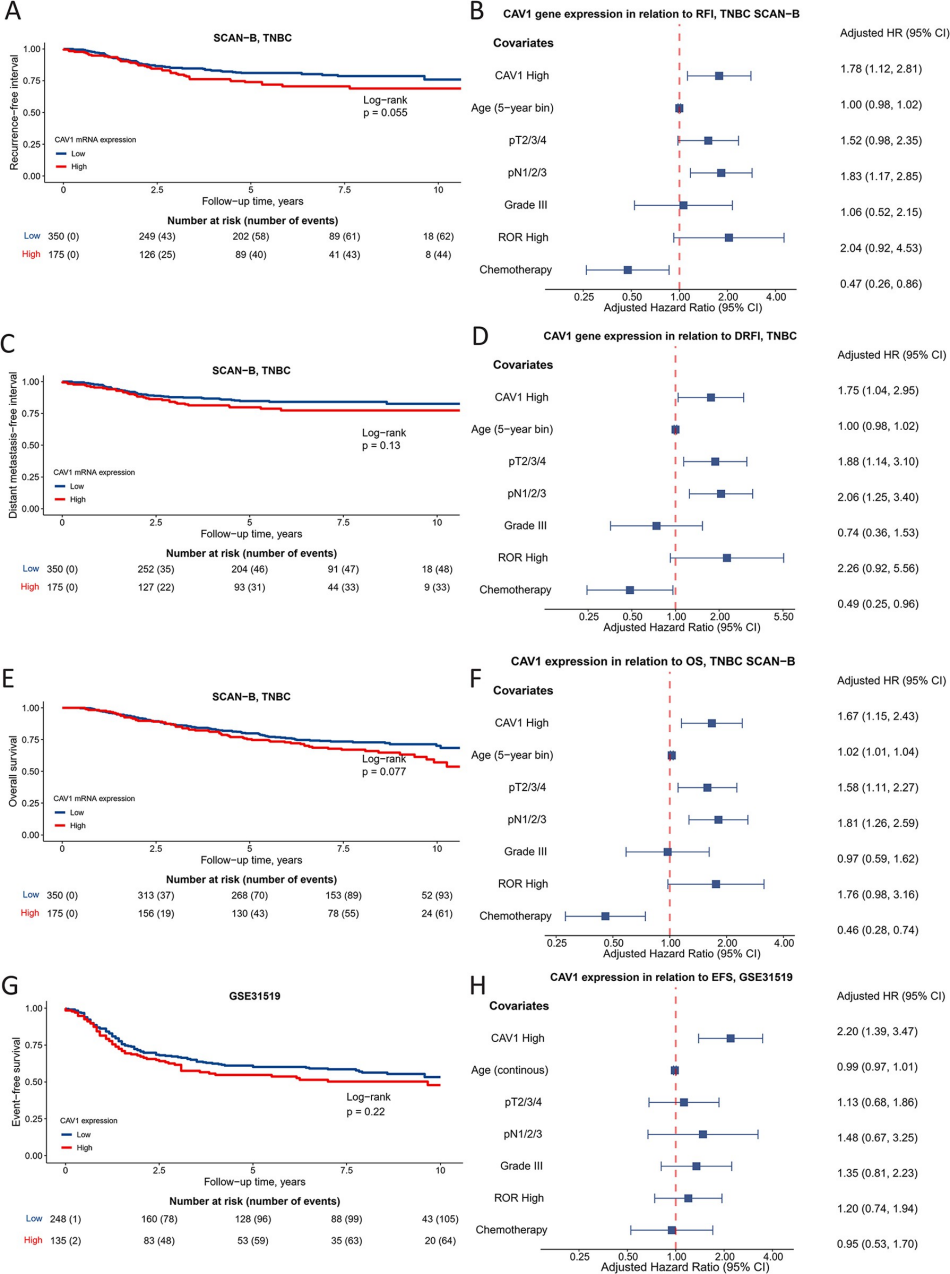
Univariable and multivariable survival analyses of *CAV1* expression. (**A**) Kaplan-Meier estimates of *CAV1* expression (dichotomous) in relation to recurrence-free interval and (**B**) corresponding forest plots of mutually adjusted hazard ratios (95% confidence intervals) in SCAN-B GEX. (**C**) Kaplan-Meier estimates of *CAV1* expression (dichotomous) in relation to distant metastasis-free interval and (**D**) corresponding forest plots of mutually adjusted hazard ratios (95% confidence intervals) in SCAN-B GEX. (**E**) Kaplan-Meier estimates of *CAV1* expression (dichotomous) in relation to overall survival and (**F**) corresponding forest plots of mutually adjusted hazard ratios (95% confidence intervals) in SCAN-B GEX. (**G**) Kaplan-Meier estimates of *CAV1* expression (dichotomous) in relation to event-free survival and (**H**) corresponding forest plots of mutually adjusted hazard ratios (95% confidence intervals) in GSE31519. The number of patients is indicated at each time point.

In the univariable survival analyses of the TNBC cohorts with *CAV1* expression, there was some evidence that *CAV1-*high in SCAN-B was associated with increased incidence of recurrence HR 1.46 (95% CI 0.99–2.14), distant metastasis HR 1.40 (95% CI 0.90–2.18), and death HR 1.33 (95% CI 0.97–1.84), Fig 4A, 4C, 4E. However, in the multivariable analyses adjusted for age, clinicopathological factors, and treatment, the evidence was stronger for an association; *CAV1-*high in SCAN-B conferred an increased incidence of recurrence HR 1.78 (95% CI 1.12–2.81), distant metastasis HR 1.75 (95% CI 1.04–2.95), and death HR 1.67 (95% CI 1.15–2.43), Fig 4B, 4D, 4F. Likewise, in the GSE31915 cohort, *CAV1-*high was not associated with EFS, HR 1.21 (95% CI 0.89–1.66) in the univariable analysis, whereas much stronger evidence for an association was seen in the multivariable analysis HR 2.20 (95% CI 1.39–3.47), Fig 4G, 4H. Interestingly, adding the PAM50 ROR score to *CAV1* expression further improved the Cox regression model’s ability to predict distant metastasis but not when solely adding the PAM50 ROR score to standard clinical models, Table 2. This improvement in predictive ability was seen in SCAN-B but not in GSE31519. In contrast to the more recent cohorts, *CAV1* expression in TNBC in METABRIC was not associated with clinical outcome in either univariable or multivariable survival analyses.

**Table 2:** Comparison of clinical prediction models of distant metastasis with or without PAM50 and *CAV1* expression using Akaike information criteria (AIC).

**SCAN-B**					
Cox regression models	No. of variables	AIC (corrected)	Delta	Akaike weights	Log-likelihood
Clinical Model + PAM50 + *CAV1* expression	7	978.50	0.00	0.54	"-482.12"
Clinical Model + CAV1 expression	6	979.87	1.37	0.27	"-483.84"
Clinical Model	5	981.74	3.24	0.11	"-485.80"
Clinical Model + PAM50 ROR	6	982.14	3.64	0.09	"-484.97"
**GSE31519**					
Cox regression models	No. of variables	AIC (corrected)	Delta	Akaike weights	Log-likelihood
Clinical Model + *CAV1* expression	6	839.27	0.00	0.68	"-413.45"
Clinical Model + PAM50 + *CAV1* expression	7	840.85	1.58	0.31	"-413.18"
Clinical Model	5	847.74	8.47	0.01	"-418.74"
Clinical Model + PAM50 ROR	6	849.77	10.50	0.00	"-418.70"

Clinical Model: Age (5-year bin), Tumor size, Nodal status, Grade, Chemotherapy

AIC: Akaike information criteria

## Discussion

In this study, we report that high *CAV1* gene expression was an independent predictor of inferior survival in patients with TNBC in three large cohorts after adjustment for clinical predictors and treatment. In addition, molecular features related to *CAV1* gene expression indicate a potential role of CAV1 in tumor vasculature that supports altered metabolism, neovascularization, and EMT combined with suppressed immune response. This may provide putative biological explanations for the observed negative impact of *CAV1* gene expression on clinical outcome. This study is, to our knowledge, the largest and most comprehensive to date investigating CAV1 in relation to molecular characteristics, tumor microenvironment composition, and prognosis in TNBC.

The consistent correlation across cohorts with the lipid module indicates a connection between CAV1 and lipid metabolism in breast cancer, which is in line with other studies [44]. It is known that CAV1 ensures the availability of the lipids required for maintaining the membrane integrity of tumor cells and modulates lipid metabolism and fatty acid oxidation [44]. For instance, loss of CAV1 leads to impaired lipid storage, lipid droplet formation, and downregulation of lipid metabolic processes *in vivo* and *in vitro* [44]. Several studies show that CAV1 is also involved in the modulation of glycolytic activities (also known as the Warburg effect), which is key for tumor survival [44]. High *CAV1* expression can stimulate glucose transporter 3 (GLUT3) transcription, increasing glucose uptake and ATP production [45]. Knockdown of CAV1 reduces cellular glucose uptake and lactate output, which would indicate suppression of the Warburg effect [45]. Other studies have shown that CAV1 interacts with insulin- and IGF-1 receptors (IR/IGF-1R) and stimulates IR/IGF-1R signaling, which enhances glucose uptake and lactate output through AKT signaling [46]. The results of DGE and GSEA analyses and the correlation between *CAV1* gene expression and early response to growth factors module support the hypothesis that CAV1 interacts with IR/IGF-1R signaling and enables metabolic alterations in the tumors, which enhances survival. Seemingly, CAV1 is involved in the regulation of the switch between glucose dependent mitochondrial respiration and aerobic glycolysis and lipid-dependent energy metabolism needed for tumor survival [47]. Further characterization of CAV1 in the metabolic context of TNBC and its TME is needed.

Furthermore, we report that CAV1 was highly expressed in endothelial cells and linked to angiogenesis, platelet activation, and abundance of endothelial cells. It is well-known that tumors rely on (neo)vascularization to survive and fulfill their metabolic needs [15]. Studies suggest that CAV1 has a role in the modulation of ischemic angiogenesis through the regulation of vascular endothelial growth factor (VEGF) dependent endothelial nitric oxide synthase (eNOS) activation in endothelial cells [48]. Ischemia is strongly linked to hypoxia where CAV1 is clearly implicated [12–14]. Hypoxia-inducible factors 1α and 2α (HIF1α and 2α) directly target CAV1 as a transcriptional target that, in turn, induces metabolic reprogramming through attenuation of MYC expression [49]. The downregulation of MYC response was also seen in the GSEA results in our study. Furthermore, endothelial cells have key immunomodulatory properties in the anti-tumor response mediated by immune cells through the regulation of extravasation and exclusion of immune cells entering the tumor via the bloodstream [15, 16, 50]. Potentially, this could explain the association between high *CAV1* expression and immunodeficiency in the TME observed in our study. The tumor vasculature is also key for the promotion of metastasis, with intravasation of malignant cells being a key event required for metastasis [15, 16, 50]. However, this process is not fully characterized. Furthermore, it is unknown how CAV1 is related to this process, and the topic merits further study.

CAV1 was highly expressed in stromal cells, which is in line with other studies [12–14]. The association between normal-like, mesenchymal stem-like, Stroma module and *CAV1* gene expression also indicates a strong connection to stromal cells and an active TME, in line with the previous literature [12–14]. CAV1 expression was especially high in *CXCL12* endothelial cells. CXCL12 is important for endothelial–fibroblast crosstalk, which is necessary for angiogenesis, tumor growth, and intravasation [51]. In addition, *CAV1* expression was correlated to endothelial cell states (S01 and S02), which are also implicated in neovascularization and angiogenesis [52], providing additional support for the role of CAV1 in tumor-related angiogenesis. The type of PVLs in which *CAV1* was highly expressed are also enriched in stem cell markers and platelet-derived growth factor activity [40]. In glioma and prostate cancer, CAV1 has been implicated as a perquisite for maintaining tumor stemness, where it is known to have a regulatory role in platelet-derived growth factor signaling [53–55]. CAFs enriched for EMT features and myogenesis were associated with *CAV1* expression in the single-cell atlas of human breast cancers and the ECOTYPER derived cell states in SCAN-B, [40, 41], potentially facilitating metastasis of TNBC. *CAV1* expression was associated with CE2 implicated in extracellular-matrix-related remodeling and fibrosis [56], supporting the hypothesis that CAV1 can remodel the surrounding extra cellular matrix [57], thus excluding immune cells and promoting metastasis. CAV1 was also highly correlated with CE6, an ecosystem characterized by the enrichment of stromal features and cells that has been reported to be associated with worse prognosis features in gastric cancer [58].

Surprisingly, *CAV1* gene expression was not correlated to CAV1 protein expression in tumors, in contrast to what we have previously reported based on TCGA data [17]. It should be noted that the previous study measured CAV1 protein expression using a reverse-phase protein array [17] and not IHC, which is a relatively crude method but is easily translated into the clinic. With the advent of IHC spatial analysis software that provides fine-tuned estimates of protein expression, some correlations may be found. Another difference from most studies that mainly evaluated CAV1 expression in bulk tissue is that in this study, we evaluated CAV1 in different spatial localizations (malignant cells and stromal cells) of the tumors; however, CAV1 in endothelial cells was not evaluated. CAV1 is abundantly expressed in endothelial cells [12–14], which was also seen in our results. Incorporating the protein expression of CAV1 in endothelial cells into the correlation analyses likely would have yielded stronger correlations. However, we were unable to properly assess CAV1 staining in the endothelium since we had access only to TMA cores, where the endothelium was often missing. Another limitation of this study is the absence of IHC markers used to differentiate between tumor and stromal cells, so it relied instead on morphological assessment as per previous studies [17–19, 25, 59]. Simultaneously utilizing multiple IHC markers allows for the subtyping of tumor and stromal cells, potentially revealing associations between CAV1 protein expression and clinical outcomes specific to particular subsets of tumor and stromal cells. However, the subtyping of tumor and stromal cells was outside the scope of this study. Additionally, a recent investigation of EHD2, a caveolar regulatory protein, highlighted that its expression in the nucleus or cytoplasm had differing associations with survival [60]. However, despite these findings, nuclear staining of CAV1 could not be detected in our study; this difference might be due to the use of a different CAV1 antibody [60]. Notably, *EHD2* and *CAV1* mRNAs were found to be coordinately expressed and jointly associated with shorter survival in basal breast cancer [60]. Regardless, the results of the present study must be interpreted in the context of the biological phenotype related to high *CAV1* mRNA expression.

CAV1 has been shown to modulate the treatment efficacy of chemotherapy (including anthracyclines and taxanes) in breast cancer in both preclinical and clinical settings [9, 10, 25, 61]. Potentially, tumors with high *CAV1* expression are more chemoresistant and respond poorly to taxane-based chemotherapy, as previously reported in the GeparSepto trial [11]. This could explain why patients with TNBC tumors having high *CAV1* expression have inferior survival since chemotherapy was the only systemic treatment offered to these patients. It may also explain why the *CAV1* gene expression was not prognostic in METABRIC due to the chemotherapy regimen being different in METABRIC (cyclophosphamide, methotrexate, and fluorouracil-based regimen) compared to SCAN-B and GSE31519 (anthracycline and taxane-based regimen). Since our study is based on real world data but does not include any data from randomized clinical trials, it is not possible to validate *CAV1* gene expression as a potential treatment-predictive biomarker for taxane-based chemotherapy. It would therefore be of great interest to further elucidate whether *CAV1* gene expression could be used as a treatment-predictive biomarker for taxane-based chemotherapy in the clinical setting. Furthermore, *in vitro* and *in vivo* studies investigating the synergism between targeted CAV1 therapy (e.g., statins) and chemotherapy could lend further credence to CAV1 as a drug target in TNBC [61, 62].

In contrast to SCAN-B, METABRIC and GSE31519 are not population-based [63]. Since tumors in METABRIC and GSE31519 were from patients with more advanced disease who were more likely to be treated at tertiary centers and to be included in clinical trials, the underlying risk of recurrence and death is considerably higher than in SCAN-B, making direct comparisons regarding prognosis harder. The SCAN-B GEX cohort is larger than both METABRIC and GSE31519; hence, the ability of the statistical test to detect potential survival associations is smaller in the latter cohorts. It should also be mentioned that the cut-offs for *CAV1-*high and *CAV1*-low classifications of TNBC are relative to a population and not based on absolute cut-offs for each tumor. The classifications were applied separately for each cohort, meaning that some tumors would be reclassified if a uniform cut-off had been applied [63].

SCAN-B is, to our knowledge, the largest breast cancer cohort to date with available RNA-seq data for consecutively enrolled breast cancers. The cohort offers unique advantages in that it allows for the evaluation of biomarkers in a contemporary, real-world setting [21–23]. The study cohort can be considered representative of the general patient demographics in the catchment area [21–23]. All herein investigated cohorts have relatively long follow-ups with median follow-up exceeding five years. Most recurrences occur within five years for TNBC [1–3]. Less than 5% of patients with TNBC have a recurrence after five years [64]. To our knowledge, this study is the most comprehensive molecular characterization of *CAV1* gene expression in TNBC and the only study that investigates the role of CAV1 in the tumor microenvironment. Further, the associations with molecular features are stable across the diverse set of investigated cohorts, and the survival associations are replicated in two independent cohorts, validating the role of *CAV1* gene expression as a prognostic marker in TNBC.

In conclusion, our findings show that high *CAV1* gene expression is an independent factor of poor prognosis in TNBC. The putative role of CAV1 in chemoresistance and a tumor-promoting TME, corroborated by molecular features, may explain this finding. Hence, *CAV1* expression is a biomarker that merits further investigation in clinical trials and as a therapeutic target.

## Supporting information

S1 File Contains S1-S7 Figs with figure legends for each figure provided below: **S1 Fig**. Flowchart of included and excluded patients in SCAN-B TMA. **S2 Fig**. *CAV1* expression by PAM50 and ROR category. *CAV1* expression (continuous) by PAM50 molecular subtype in (**A**) SCAN-B GEX, (**B**) GSE31519, and (**C**) METABRIC. *CAV1* expression (continuous) by PAM50 ROR category in (**D**) SCAN-B GEX, (**E**) GSE31519, and (**F**) METABRIC. **S3 Fig**. CAV1 protein levels in different spatial localizations in relation to molecular features and *CAV1* gene expression. CAV1 protein levels in (**A**) malignant cells and in (**B**) stromal cells by PAM50 molecular subtype in SCAN-B TMA. CAV1 protein levels in (**C**) malignant cells and in (**D**) stromal cells by TNBC molecular subtype in SCAN-B TMA. *CAV1* gene expression in relation to CAV1 protein levels in (**E**) malignant cells, (**F**) stromal cells, and (**G**) combined protein status. **S4 Fig**. Molecular analyses of *CAV1* expression in SCAN-B. **(A)** Volcano plot showing up- and downregulated genes in *CAV1-*high compared to *CAV1-*low tumors. (**B**) Dot plots showing activated and suppressed. (**C**) Hallmark signatures and GO terms in *CAV1-*high compared to *CAV1-*low tumors. (**D**) Heatmap of differentially expressed genes (DEG) in *CAV1-*high compared to *CAV1-*low tumors. **S5 Fig**. *CAV1* gene expression in different cell populations in the single-cell atlas of human breast cancers. Log-normalized expression of *CAV1* in (**A**) a Uniform Manifold Approximation and Projection (UMAP) visualization of different breast cancer cells and (**B**) corresponding violin plots. Log-normalized expression of *CAV1* in (**C**) a UMAP visualization of major subtypes of stromal cells in breast cancer and (**D**) corresponding violin plots. **S6 Fig**. Tumor microenvironment composition in relation to *CAV1* gene expression. Log-normalized expression of *CAV1* in (**A**) a UMAP visualization of specialized subtypes of stromal cells in breast cancer and (**B**) corresponding violin plots. Relative abundance of (**C**) fibroblasts and (**D**) endothelial cells in *CAV1-*high and low tumors. (**E**) Pearson correlations of *CAV1* gene expression (continuous) and the different fibroblast cell states. (**F**) Pearson correlations of *CAV1* gene expression (continuous) and the different endothelial cell states. (**G**) The dominant CE in *CAV1-*high and *CAV1*-low tumors. (**H**) Pearson correlations of *CAV1* gene expression (continuous) and relative abundance of the CE. **S7 Fig**. CAV1 protein levels in different spatial localizations in relation to clinical outcomes. Kaplan-Meier estimates of CAV1 protein levels in (**A**) malignant cells and (**B**) stromal cells in relation to recurrence-free interval in SCAN-B TMA. CAV1 protein levels in (**C**) malignant cells and (**D**) stromal cells in relation to distant metastasis-free interval in SCAN-B TMA. CAV1 protein levels in (**E**) malignant cells and (**F**) stromal cells in relation to overall survival in SCAN-B TMA. The number of patients is indicated at each time-point.
(PDF)

S2 File Contains S1-S3 and S7 Tables provided in a word file. (DOCX)

S3 File Contains S4-S6 Tables provided in an excel file. (XLSX)

## Abbreviations

AIC: Akaike information criteria
BCSS: Breast cancer-specific survival
CAF: Cancer-associated fibroblast
CAV1: Caveolin-1
DEGs: Differentially expressed genes
DGE: Differential gene expression
DMFI: Distant metastasis-free interval
EGFR: Epidermal growth factor receptor
ER: Estrogen receptor
EMT: Epithelial-mesenchymal transition
FC: Fold change
FDR: False discovery rate
FPKM: Fragments per kilobase of exon per million mapped reads
GEX: Gene expression profile
GO: Gene Ontology
GOBO: Gene expression-based Outcome for Breast cancer Online
GSEA: Gene set enrichment analysis
HER2: Human epidermal growth factor receptor 2
HIF1α: Hypoxia-inducible factor 1 α
HIF2α: Hypoxia-inducible factor 2 α
HR: Hazard ratio
IHC: Immunohistochemistry
ISH: In situ hybridization
OS: Overall survival
METABRIC: Molecular Taxonomy of Breast Cancer International Consortium
PARP: Poly (ADP-ribose) polymerase
PR: Progesterone receptor
RFI: Recurrence-free interval
ROR: Risk of Recurrence
RNA-seq: Massive parallel paired-end sequencing of mRNA
SCAN-B: Swedish Cancerome Analysis Network—Breast
TGFβ: Transforming growth factor beta
TME: Tumor microenvironment
TNBC: Triple-negative breast cancer
TROP2: Tumor-associated calcium signal transducer 2
UMAP: Uniform Manifold Approximation and Projection
VEGF: Vascular endothelial growth factor
