# Correction: High Caveolin-1 mRNA expression in triple-negative breast cancer is associated with an aggressive tumor microenvironment, chemoresistance, and poor clinical outcome

**DOI:** 10.1371/journal.pone.0322403

**Published:** 2025-04-08

**Authors:** Christopher Godina, Somayeh Khazaei, Mattias Belting, Johan Vallon-Christersson, Björn Nodin, Karin Jirström, Karolin Isaksson, Ana Bosch, Helena Jernström

The S4 and S5 Figs are missing in S1 File. Please see the correct S1 File here.

## Supporting Information

S1 File Supplementary dataset.
This file includes supplementary data.
(PDF)
