# Supplementary material for: High Caveolin-1 mRNA expression in triple-negative breast cancer is associated with an aggressive tumor microenvironment, chemoresistance, and poor clinical outcome
Source: PLoS One. 2024 Jul 3;19(7):e0305222. doi: 10.1371/journal.pone.0305222 (PMC11221642; doi:10.1371/journal.pone.0305222)
Supplement: S2 File — (DOCX) [file pone.0305222.s002.docx]

**High caveolin-1 expression in triple-negative breast cancer is associated with an aggressive tumor microenvironment, chemoresistance and poor clinical outcome**

Christopher Godina, Somayeh Khazaei, Mattias Belting, Johan Vallon-Christersson, Björn Nodin, Karin Jirström, Karolin Isaksson, Ana Bosch, Helena Jernström.

Table of Contents

S1 Table………………………………………………………………………2

S2 Table………………………………………………………………………3

S3 Table………………………………………………………………………4

S7 Table………………………………………………………………………5

S4-6 Tables are available separately as an .xlsx file (S3 File).

| **S1 Table.** CAV1 protein levels in malignant and stromal cells in relation to clinicopathological factors | | | | | | |
| --- | --- | --- | --- | --- | --- | --- |
|  | All  patients | Miss-  ing | CAV1 in malignant cells  n= 231 | | CAV1 in stromal cells  n= 227 | |
|  |  |  | Negative to moderate | Strong | Negative to moderate | Strong |
|  | n=242 |  | n= 143 | n=88 | n=108 | n=119 |
|  | Number (%) |  | Number (%) | Number (%) | Number (%) | Number (%) |
|  | Median (IQR) |  | Median (IQR) | Median  (IQR) | Median  (IQR) | Median  (IQR) |
| **Age at diagnosis, years** | 61 (50─72) | 0 | 63 (51─72) | 61 (47.5─72.5) | 58 (48─69.5) | 65 (51─724) |
| –40 | 31 (13.8) |  | 19 (14.1) | 9 (11.4) | 16 (15.5) | 10 (9.3) |
| 41–50 | 28 (12.4) |  | 13 (9.6) | 14 (17.7) | 14 (13.6) | 13 (12.1) |
| 51–60 | 51 (22.7) |  | 31 (23.0) | 15 (19.0) | 28 (27.8) | 19 (17.8) |
| 61–70 | 58 (25.8) |  | 37 (27.4) | 20 (25.3) | 22 (21.4) | 32 (29.9) |
| 71–80 | 30 (13.3) |  | 20 (14.8) | 9 (11.4) | 12 (11.7) | 17 (15.9) |
| 81– | 27 (12.0) |  | 15 (11.1) | 12 (15.2) | 11 (10.7) | 16 (15.0) |
| **Invasive tumor size** |  | 2 |  |  |  |  |
| pT2/3/4 | 109 (45.4) |  | 63 (44.4) | 45 (51.7) | 46 (43.0) | 59 (50.0) |
| **Axillary lymph node involvement** |  | 6 |  |  |  |  |
| pN1/2/3 (any) | 81 (34.3) |  | 58 (41.1) | 20 (23.5) | 28 (26.9) | 50 (42.4) |
| **Main histological type** |  | 0 |  |  |  |  |
| No special type (formerly ductal) | 211 (87.2) |  | 120 (83.9) | 82 (93.2) | 98 (90.8) | 101 (84.9) |
| Lobular | 8 (3.3) |  | 6 (4.2) | 1 (1.14) | 3 (2.8) | 4 (3.4) |
| Other or mixed | 23 (9.5) |  | 17 (11.9) | 5 (5.7) | 7 (6.5) | 14 (11.8) |
| **Histological grade** |  | 3 |  |  |  |  |
| I | 0 |  | 0 | 0 | 0 | 0 |
| II | 33 (13.8) |  | 31 (21.8) | 2 (2.3) | 6 (5.6) | 27 (22.7) |
| III | 209 (86.2) |  | 111 (78.2) | 86 (97.7) | 101 (94.4) | 92 (77.3) |
| **Systemic Treatment** |  |  |  |  |  |  |
| Chemotherapy | 202 (83.8) | 1 | 118 (82.5%) | 73 (83.9%) | 91 (84.3) | 96 (81.4) |
| **PAM50 Subtype** |  | 0 |  |  |  |  |
| Luminal A | 1 (0.4) |  | 0 (0.0) | 1 (1.3) | 1 (1.0) | 0 (0.0) |
| Luminal B | 1 (0.4) |  | 1 (0.7) | 0 (0.0) | 1 (1.0) | 0 (0.0) |
| Normal-like | 28 (12.4) |  | 24 (17.8) | 4 (5.1) | 7 (6.8) | 21 (19.6) |
| HER2 enriched | 29 (12.9) |  | 26 (19.3) | 2 (2.5) | 3 (2.9) | 24 (22.4) |
| Basal | 166 (73.8) |  | 84 (62.2) | 72 (91.1) | 91 (88.3) | 62 (57.9) |
| **PAM50 ROR** |  | 27 |  |  |  |  |
| Low | 20 (9.3) |  | 17 (12.8) | 3 (3.9) | 6 (6.0) | 14 (13.2) |
| Intermediate | 14 (6.5) |  | 10 (7.5) | 4 (5.2) | 4 (4.0) | 10 (9.4) |
| High | 181 (84.2) |  | 106 (80.0) | 70 (90.1) | 90 (90.0) | 82 (77.4) |
| **TNBC Subtype** |  | 22 |  |  |  |  |
| BL1 | 43 (19.5) |  | 18 (13.7) | 21 (26.9) | 27 (27.0) | 10 (9.5) |
| BL2 | 22 (10.0) |  | 12 (9.2) | 8 (10.3) | 9 (9.0) | 10 (9.5) |
| IM | 41 (18.6) |  | 30 (22.9) | 8 (10.3) | 23 (23.0) | 15 (14.3) |
| LAR | 29 (13.2) |  | 27 (20.6) | 2 (2.6) | 3 (3.0) | 26 (24.8) |
| M | 40 (18.2) |  | 11 (13.9) | 22 (28.2) | 21 (21.0) | 18 (17.1) |
| MSL | 17 (7.7) |  | 11 (8.4) | 5 (6.4) | 7 (7.0) | 9 (8.6) |
| UNS | 28 (12.7) |  | 16 (12.2) | 12 (15.4) | 10 (10.0) | 17 (16.2) |

| **S2 Table.** Descriptive statistics of *CAV1-*high and low tumors in METABRIC | | | | |
| --- | --- | --- | --- | --- |
|  | METABRIC n = 320 | | | |
|  | All | Miss-ing | *CAV1* mRNA expression  n= 320 | |
|  | patients |  | Low | High |
|  | n=320 |  | n=214 | n=106 |
|  | Number (%) |  | Number (%) | Number (%) |
|  | Median (IQR) |  | Median (IQR) | Median (IQR) |
| **Age at diagnosis, years** | 61.8 (51.4–70.6) | 0 | 55.0 (44.7–65.7) | 57.4 (45.7–66.5) |
| –40 | 52 (16.2) |  | 34 (15.9) | 18 (17.0) |
| 41–50 | 72 (22.5) |  | 52 (24.3) | 20 (18.9) |
| 51–60 | 82 (25.6) |  | 55 (25.7) | 27 (25.5) |
| 61–70 | 72 (22.5) |  | 45 (21.0) | 27 (25.5) |
| 71–80 | 33 (10.3) |  | 21 (9.8) | 12 (11.3) |
| 81– | 9 (2.8) |  | 7 (3.3) | 2 (1.9) |
| **Invasive tumor size** |  | 6 |  |  |
| pT2/3/4 | 184 (58.6) |  | 129 (61.1) | 55 (53.4) |
| **Axillary lymph node involvement** |  | 0 |  |  |
| pN1/2/3 (any) | 162 (50.6) |  | 118 (55.1) | 44 (41.5) |
| **Main histological type** |  | 10 |  |  |
| No special type (formerly ductal) | 262 (84.5) |  | 182 (86.3) | 80 (80.8) |
| Lobular | 15 (4.8) |  | 10 (4.7) | 5 (5.1) |
| Other or mixed | 33 (10.6) |  | 19 (9.0) | 14 (14.1) |
| **Histological grade** |  | 8 |  |  |
| I | 4 (1.3) |  | 2 (0.9) | 2 (2.0) |
| II | 40 (12.8) |  | 18 (8.5) | 22 (21.8) |
| III | 268 (85.9) |  | 191 (90.5) | 77 (76.2) |
| **Systemic Treatment** |  | 0 |  |  |
| Chemotherapy | 165 (51.6) |  | 117 (54.7) | 48 (45.3) |
| **PAM50 Subtype** |  | 0 |  |  |
| Luminal A | 2 (0.6) |  | 0 (0.0) | 2 (1.9) |
| Luminal B | 0 (0.0) |  | 0 (0.0) | 0 (0.0) |
| Normal-like | 25 (7.8) |  | 3 (1.4) | 22 (20.8) |
| HER2 enriched | 0 (0.0) |  | 32 (15.0) | 7 (6.6) |
| Basal | 254 (79.4) |  | 179 (83.6) | 75 (70.8) |
| **PAM50 ROR** |  | 5 |  |  |
| Low | 22 (7.0)) |  | 4 (1.9) | 18 (17.5) |
| Intermediate | 55 (17.5) |  | 28 (13.2) | 27 (26.2) |
| High | 238 (75.6) |  | 180 (84.9) | 58 (56.3) |
| **TNBC Subtype** |  | 0 |  |  |
| BL1 | 72 (22.5) |  | 61 (28.5) | 11 (10.4) |
| BL2 | 22 (6.9) |  | 18 (8.4) | 4 (3.8) |
| IM | 65 (20.3) |  | 53 (24.8) | 12 (11.3) |
| LAR | 39 (12.2) |  | 29 (13.6) | 10 (9.4) |
| M | 48 (15.0) |  | 27 (12.6) | 21 (19.8) |
| MSL | 38 (11.9) |  | 2 (0.9) | 36 (34.0) |
| UNS | 36 (11.2) |  | 24 (11.2) | 12 (11.3) |

| **S3 Table.** Descriptive statistics of *CAV1-*high and low tumors in GSE31519 | | | | |
| --- | --- | --- | --- | --- |
|  | GSE31519 n = 579 | | | |
|  | All  patients | Miss-  ing | *CAV1* mRNA expression  n= 579 | |
|  |  |  | Low (T1/T2) | High (T3) |
|  | n=579 |  | n=386 | n=193 |
|  | Number (%) |  | Number (%) | Number (%) |
|  |  |  |  |  |
| **Age at diagnosis, years** | ─ | 130 | ─ | ─ |
| –40 | 88 (19.6) |  | 61 (21.0) | 27 (17.1) |
| 41–50 | 132 (29.4) |  | 89 (30.6) | 43 (27.2) |
| 51–60 | 115 (25.6) |  | 76 (26.1) | 39 (24.7) |
| 61–70 | 73 (16.3) |  | 42 (14.4) | 31 (19.6) |
| 71–80 | 34 (7.6) |  | 18 (6.2) | 16 (10.1) |
| 81– | 7 (1.6) |  | 5 (1.7) | 2 (1.3) |
| **Invasive tumor size** |  | 119 |  |  |
| pT2/3/4 | 346 (75.2) |  | 237 (78.2) | 109 (69.4) |
| **Axillary lymph node involvement** |  | 175 |  |  |
| pN1/2/3 (any) | 128 (31.7) |  | 88 (33.7) | 40 (28.0) |
| **Histological grade** |  | 67 |  |  |
| I/II | 128 (27.5) |  | 76 (24.4) | 52 (33.8) |
| III | 337 (72.5) |  | 235 (75.6) | 102 (66.2) |
| **Systemic Treatment** |  |  |  |  |
| Chemotherapy | 87 (24.8) | 228 | 46 (20.6) | 41 (32.0) |
| **PAM50 Subtype** |  | 0 |  |  |
| Luminal A | 67 (11.6) |  | 20 (5.2) | 41 (21.2) |
| Luminal B | 102 (17.6) |  | 71 (18.4) | 102 (17.6) |
| Normal-like | 41 (7.1) |  | 16 (4.1) | 25 (13.0)) |
| HER2 enriched | 67 (11.6) |  | 48 (12.4) | 19 (9.8) |
| Basal | 308 (53.2) |  | 231 (59.8) | 77 (39.9) |
| **PAM50 ROR** |  | 179 |  |  |
| Low | 60 (15.0) |  | 23 (8.8) | 37 (26.4) |
| Intermediate | 102 (25.5) |  | 61 (23.5) | 41 (29.3) |
| High | 238 (59.5) |  | 176 (67.7) | 62 (44.3) |
| **TNBC Subtype** |  | 68 |  |  |
| BL1 | 97 (19.0) |  | 82 (23.6) | 97 (19.0) |
| BL2 | 54 (10.6) |  | 29 (8.4) | 54 (10.6) |
| IM | 101 (19.8) |  | 84 (24.2) | 101 (19.8) |
| LAR | 53 (10.4) |  | 37 (10.7) | 53 (10.4) |
| M | 99 (19.4) |  | 63 (18.2) | 99 (19.4) |
| MSL | 42 (8.2) |  | 8 (2.3) | 42 (8.2) |
| UNS | 65 (12.7) |  | 44 (12.7) | 65 (12.7) |

| **S7 Table.** Multivariable analysis of CAV1 in malignant and stromal cells in relation to clinical outcomes in SCAN-B TMA | | | | | | | |
| --- | --- | --- | --- | --- | --- | --- | --- |
|  | **Recurrence** | | **Distant metastasis** | | | **Overall survival** | |
|  | **HR** | **(95% CI)** | **HR** | | **(95% CI)** | **HR** | **(95% CI)** |
| CAV1 (strong) in stromal cells | 0.88 | 0.46 ─ 1.69 | 0.89 | | 0.44 ─ 1.81 | 1.07 | 0.63 ─ 1.80 |
| Age (5-year bin) | 1.02 | 0.99 ─ 1.05 | 1.03 | | 0.99 ─ 1.06 | 1.05 | 1.02 ─ 1.08 |
| pT2/3/4 | 1.45 | 0.75 ─ 2.79 | 1.85 | | 0.91 ─ 3.78 | 1.50 | 0.89 ─ 2.52 |
| pN1/2/3 | 1.57 | 0.80 ─ 3.08 | 1.54 | | 0.76 ─ 3.12 | 1.26 | 0.75 ─ 2.13 |
| Grade III | 0.89 | 0.33 ─ 2.36 | 0.58 | | 0.21 ─ 1.57 | 0.83 | 0.41 ─ 1.64 |
| ROR High | 1.01 | 0.38 ─ 2.66 | 1.63 | | 0.54 ─ 4.90 | 1.36 | 0.67 ─ 2.77 |
| Chemotherapy | 0.80 | 0.31 ─ 2.03 | 1.16 | | 0.41 ─ 3.25 | 0.82 | 0.38 ─ 2.77 |
|  | **Recurrence** | | **Distant metastasis** | | | **Overall survival** | |
|  | **HR** | **(95% CI)** | **HR** | **(95% CI)** | | **HR** | **(95% CI)** |
| CAV1 (strong) in malignant cells | 0.65 | 0.31 ─ 1.36 | 0.58 | 0.26 ─ 3.32 | | 0.86 | 0.49 ─ 1.51 |
| Age (5-year bin) | 1.02 | 0.99 ─ 1.05 | 1.03 | 0.99 ─ 1.06 | | 1.05 | 1.02 ─ 1.08 |
| pT2/3/4 | 1.48 | 0.76 ─ 2.85 | 1.90 | 0.92 ─ 3.91 | | 1.49 | 0.88 ─ 2.51 |
| pN1/2/3 | 1.43 | 0.72 ─ 2.85 | 1.39 | 0.68 ─ 2.85 | | 1.26 | 0.73 ─ 2.18 |
| Grade III | 1.02 | 0.39 ─ 2.69 | 0.68 | 0.26 ─ 1.83 | | 0.85 | 0.42 ─ 1.70 |
| ROR High | 1.04 | 0.40 ─ 2.73 | 1.29 | 0.55 ─ 3.03 | | 1.35 | 0.66 ─ 2.75 |
| Chemotherapy | 0.83 | 0.32 ─ 2.11 | 1.68 | 0.57 ─ 4.97 | | 0.79 | 0.37 ─ 1.71 |
