# Supplementary material for: Correction: High Caveolin-1 mRNA expression in triple-negative breast cancer is associated with an aggressive tumor microenvironment, chemoresistance, and poor clinical outcome
Source: PLoS One. 2025 Apr 8;20(4):e0322403. doi: 10.1371/journal.pone.0322403 (PMC11978041; doi:10.1371/journal.pone.0322403)
Supplement: S1 File — This file includes supplementary data. (PDF) [file pone.0322403.s001.pdf]

High caveolin-1 expression in triple-negative breast cancer is associated with an aggressive tumor microenvironment, chemoresistance and poor clinical outcome

Christopher Godina, Somayeh Khazaei, Mattias Belting, Johan Vallon-Christersson, Björn Nodin, Karin Jirström, Karolin Isaksson, Ana Bosch, Helena Jernström.

Table of Contents

Supplementary Figure legends……………………………..…………1-2

S1 Fig……………………………………………………………………3

S2 Fig……………………………………………………………………4

S3 Fig……………………………………………………………………5

S4 Fig……………………………………………………………………6

S5 Fig……………………………………………………………………7

S6 Fig……………………………………………………………………8

S7 Fig……………………………………………………………………9

Supplementary Figure legends

S1 Fig. Flowchart of included and excluded patients in SCAN-B TMA

S2 Fig. CAV1 expression by PAM50 and ROR category

CAV1 expression (continuous) by PAM50 molecular subtype in (A) SCAN-B GEX, (B) GSE31519, and (C) METABRIC. CAV1 expression (continuous) by PAM50 ROR category in (D) SCAN-B GEX, (E) GSE31519, and (F) METABRIC.

S3 Fig. CAV1 protein levels in different spatial localizations in relation to molecular features and CAV1 gene expression

CAV1 protein levels in (A) malignant cells and in (B) stromal cells by PAM50 molecular subtype in SCAN-B TMA. CAV1 protein levels in (C) malignant cells and in (D) stromal cells by TNBC molecular subtype in SCAN-B TMA. CAV1 gene expression in relation to CAV1 protein levels in (E) malignant cells, (F) stromal cells, and (G) combined protein status.

S4 Fig. Molecular analyses of CAV1 expression in SCAN-B

(A) Volcano plot showing up- and downregulated genes in CAV1-high compared to CAV1-low tumors. (B) Dot plots showing activated and suppressed. (C) Hallmark signatures and GO terms in CAV1-high compared to CAV1-low tumors. (D) Heatmap of differentially expressed genes (DEG) in CAV1-high compared to CAV1-low tumors

S5 Fig. CAV1 gene expression in different cell populations in the single-cell atlas of human breast cancers

Log-normalized expression of CAV1 in (A) a Uniform Manifold Approximation and Projection (UMAP) visualization of different breast cancer cells and (B) corresponding violin plots. Log-normalized expression of CAV1 in (C) a UMAP visualization of major subtypes of stromal cells in breast cancer and (D) corresponding violin plots.

S6 Fig. Tumor microenvironment composition in relation to CAV1 gene expression

Log-normalized expression of CAV1 in (A) a UMAP visualization of specialized subtypes of stromal cells in breast cancer and (B) corresponding violin plots. Relative abundance of (C) fibroblasts and (D) endothelial cells in CAV1-high and low tumors. (E) Pearson correlations of CAV1 gene expression (continuous) and the different fibroblast cell states. (F) Pearson correlations of CAV1 gene expression (continuous) and the different endothelial cell states. (G) The dominant CE in CAV1-high and CAV1-low tumors. (H) Pearson correlations of CAV1 gene expression (continuous) and relative abundance of the CE.

S7 Fig. CAV1 protein levels in different spatial localizations in relation to clinical outcomes

Kaplan-Meier estimates of CAV1 protein levels in (A) malignant cells and (B) stromal cells in relation to recurrence-free interval in SCAN-B TMA. CAV1 protein levels in (C) malignant cells and (D) stromal cells in relation to distant metastasis-free interval in SCAN-B TMA. CAV1 protein levels in (E) malignant cells and (F) stromal cells in relation to overall survival in SCAN-B TMA. The number of patients is indicated at each time-point.

S1 Fig

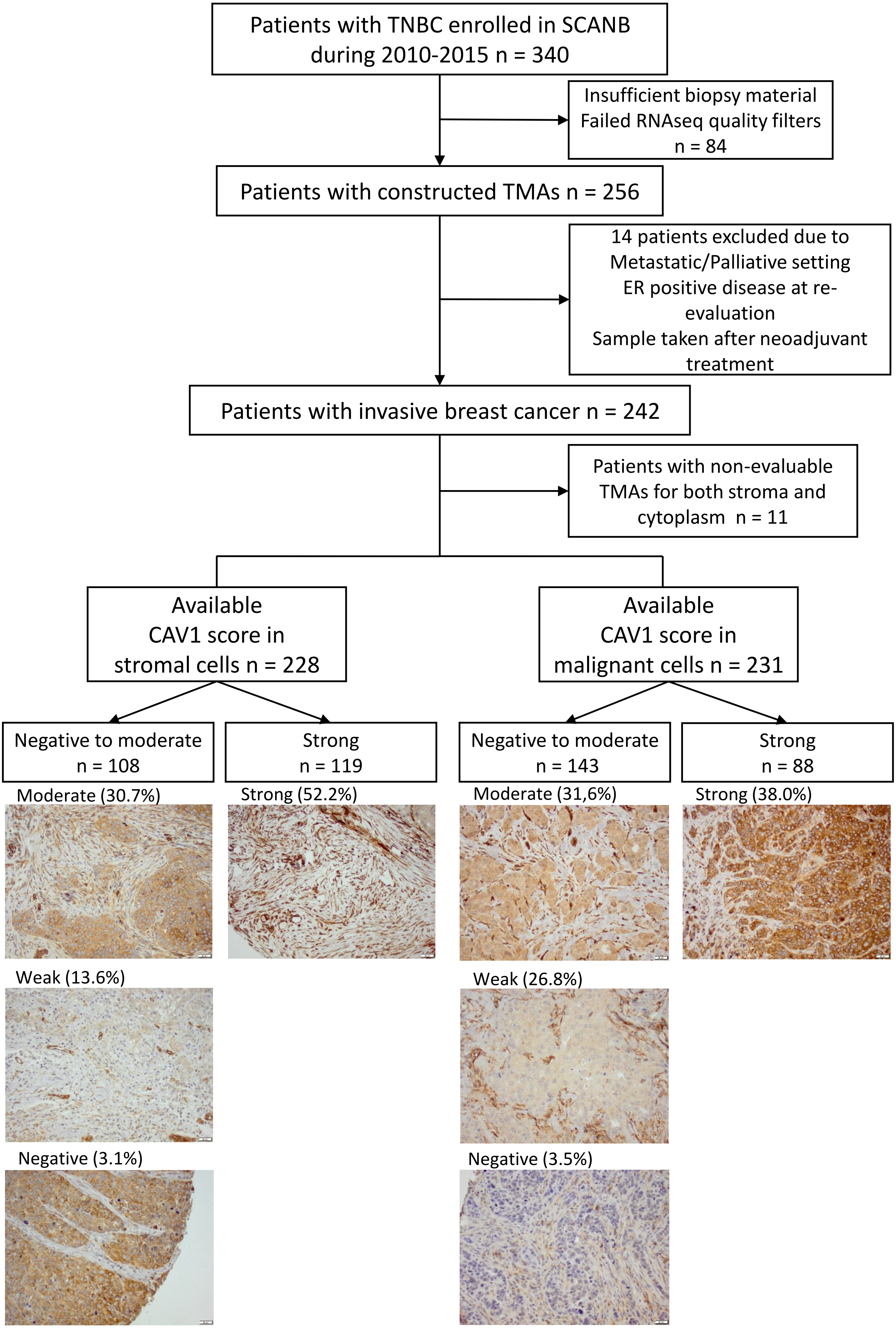
Patients with TNBC enrolled in SCANB during 2010-2015 n = 340
Insufficient biopsy material
Failed RNAseq quality filters
n = 84
Patients with constructed TMAs n = 256
14 patients excluded due to
Metastatic/Palliative setting
ER positive disease at re-evaluation
Sample taken after neoadjuvant treatment
Patients with invasive breast cancer n = 242
Patients with non-evaluable TMAs for both stroma and cytoplasm n = 11
Available CAV1 score in stromal cells n = 228
Available CAV1 score in malignant cells n = 231
Negative to moderate n = 108
Strong n = 119
Negative to moderate n = 143
Strong n = 88
Moderate (30.7%)
Strong (52.2%)
Moderate (31,6%)
Strong (38.0%)
Weak (13.6%)
Weak (26.8%)
Negative (3.1%)
Negative (3.5%)

S2 Fig

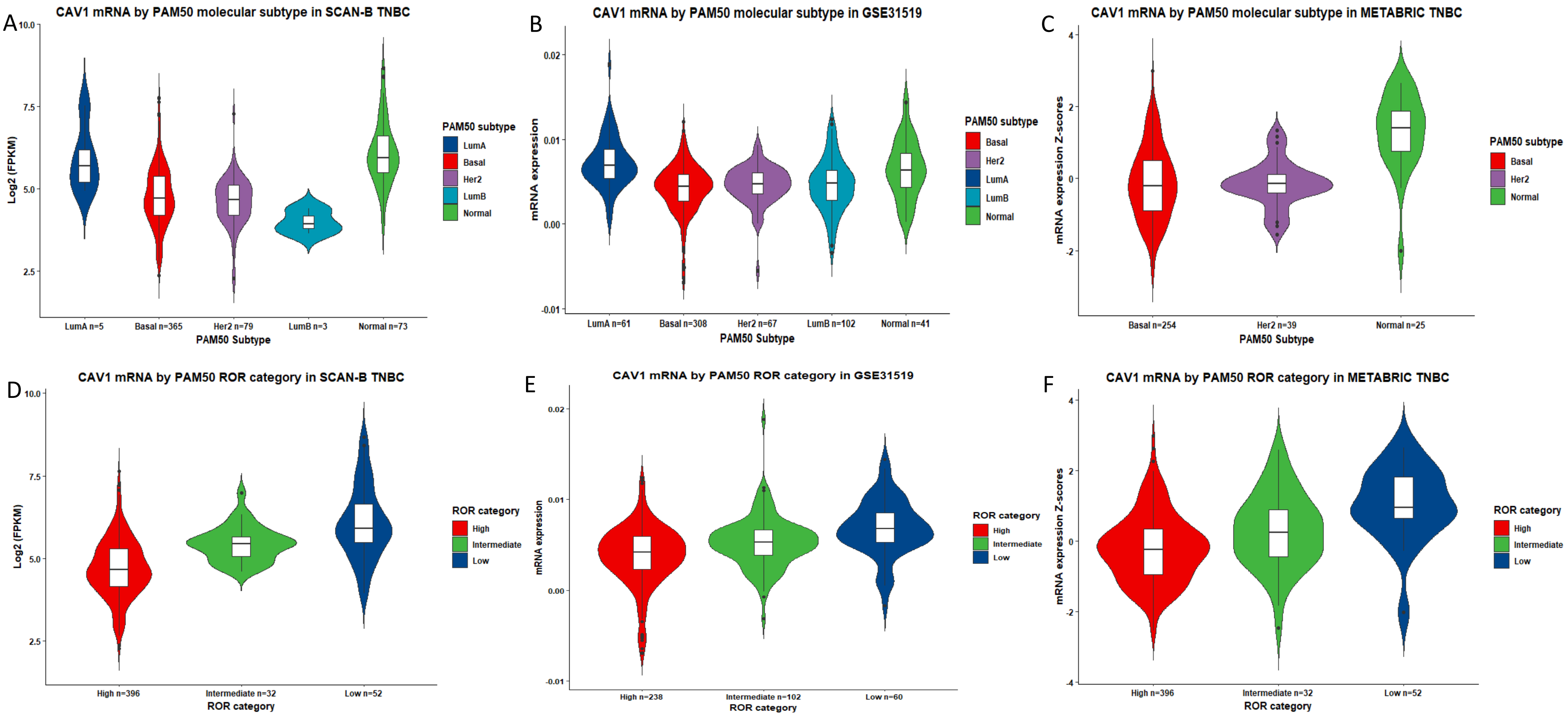
A
CAV1 mRNA by PAM50 molecular subtype in SCAN-B TNBC
Log2 (FPKM)
LumA n=5
Basal n=365
Her2 n=79
LumB n=3
Normal n=73
PAM50 Subtype
PAM50 subtype
LumA
Basal
Her2
LumB
Normal
B
CAV1 mRNA by PAM50 molecular subtype in GSE31519
mRNA expression
LumA n=61
Basal n=308
Her2 n=67
LumB n=102
Normal n=41
PAM50 Subtype
C
CAV1 mRNA by PAM50 molecular subtype in METABRIC TNBC
mRNA expression Z-scores
Basal n=254
Her2 n=39
Normal n=25
PAM50 Subtype
D
CAV1 mRNA by PAM50 ROR category in SCAN-B TNBC
Log2 (FPKM)
High n=396
Intermediate n=32
Low n=52
ROR category
High
Intermediate
Low
E
CAV1 mRNA by PAM50 ROR category in GSE31519
mRNA expression
High n=238
Intermediate n=102
Low n=60
ROR category
F
CAV1 mRNA by PAM50 ROR category in METABRIC TNBC
mRNA expression Z-scores
High n=396
Intermediate n=32
Low n=52
ROR category

S3 Fig

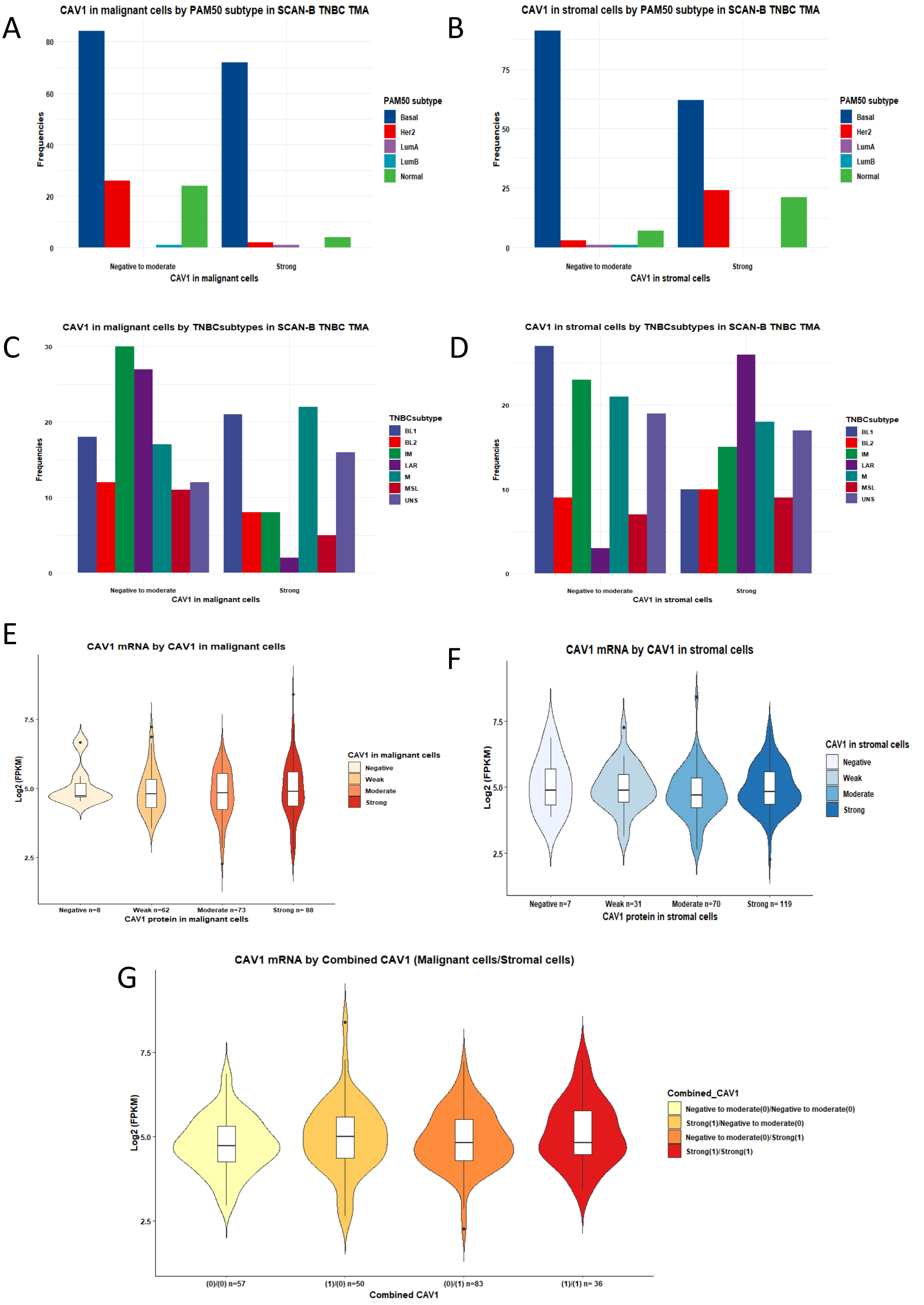
A
CAV1 in malignant cells by PAM50 subtype in SCAN-B TNBC TMA
Frequencies
80
60
40
20
0
Negative to moderate
Strong
CAV1 in malignant cells
PAM50 subtype
Basal
Her2
LumA
LumB
Normal
B
CAV1 in stromal cells by PAM50 subtype in SCAN-B TNBC TMA
Frequencies
75
50
25
0
Negative to moderate
Strong
CAV1 in stromal cells
PAM50 subtype
Basal
Her2
LumA
LumB
Normal
C
CAV1 in malignant cells by TNBCsubtypes in SCAN-B TNBC TMA
Frequencies
30
20
10
0
Negative to moderate
Strong
CAV1 in malignant cells
TNBCsubtype
BL1
BL2
IM
LAR
M
MSL
UNS
D
CAV1 in stromal cells by TNBCsubtypes in SCAN-B TNBC TMA
Frequencies
20
10
0
Negative to moderate
Strong
CAV1 in stromal cells
TNBCsubtype
BL1
BL2
IM
LAR
M
MSL
UNS
E
CAV1 mRNA by CAV1 in malignant cells
Log2 (FPKM)
7.5
5.0
2.5
Negative n=8
Weak n=62
Moderate n=73
Strong n= 88
CAV1 protein in malignant cells
CAV1 in malignant cells
Negative
Weak
Moderate
Strong
F
CAV1 mRNA by CAV1 in stromal cells
Log2 (FPKM)
7.5
5.0
2.5
Negative n=7
Weak n=31
Moderate n=70
Strong n= 119
CAV1 protein in stromal cells
CAV1 in stromal cells
Negative
Weak
Moderate
Strong
G
CAV1 mRNA by Combined CAV1 (Malignant cells/Stromal cells)
Log2 (FPKM)
7.5
5.0
2.5
(0)/(0) n=57
(1)/(0) n=50
(0)/(1) n=83
(1)/(1) n= 36
Combined CAV1
Combined_CAV1
Negative to moderate(0)/Negative to moderate(0)
Strong(1)/Negative to moderate(0)
Negative to moderate(0)/Strong(1)
Strong(1)/Strong(1)

S4 Fig

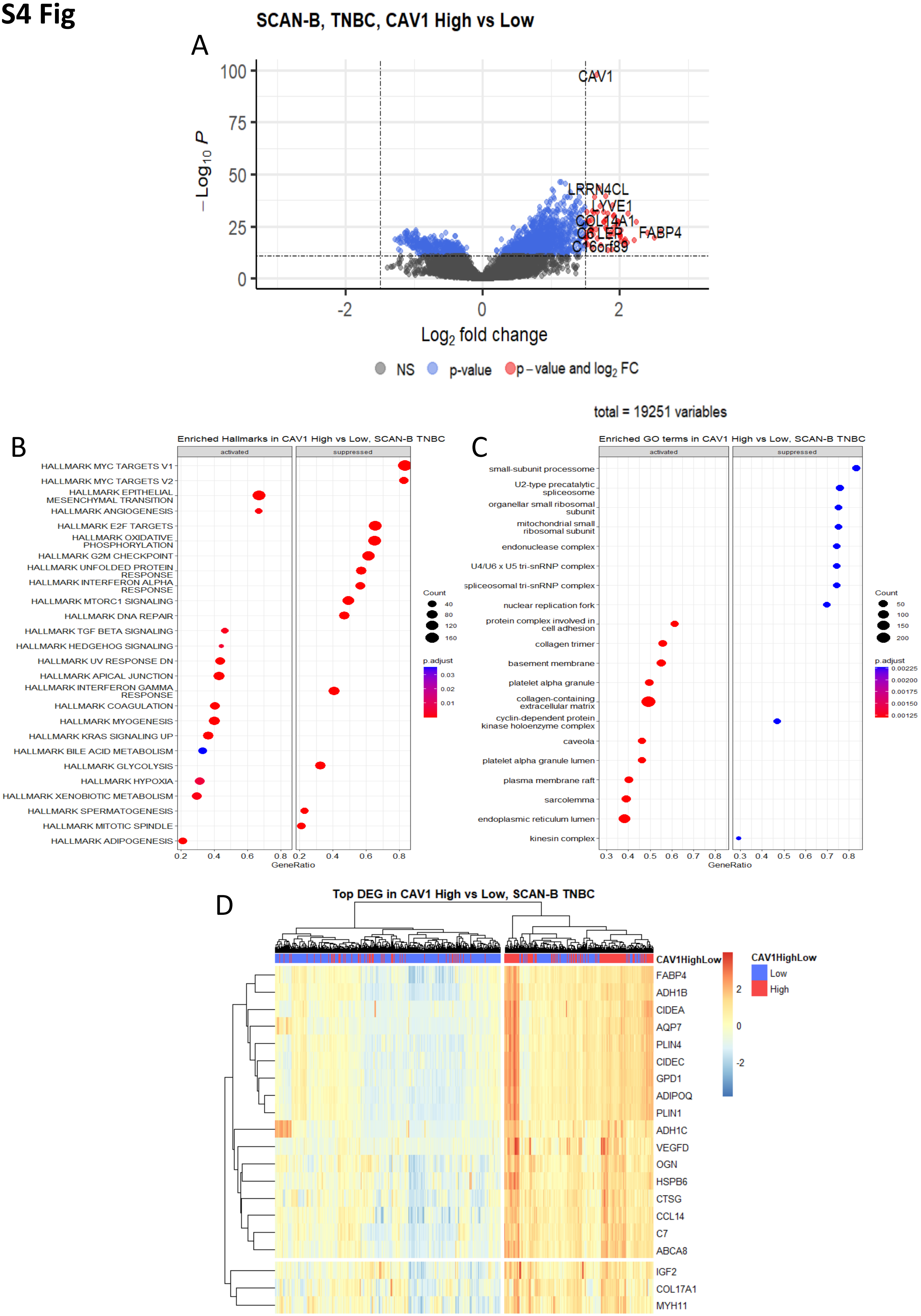

SCAN-B, TNBC, CAV1 High vs Low
A
CAV1
LRRN4CL
LYVE1
COL14A1
Q6LEP
FABP4
C16orf89
100
75
50
25
0
−Log10 P
-2
0
2
Log2 fold change
NS
p-value
p – value and log2 FC
total = 19251 variables
B
Enriched Hallmarks in CAV1 High vs Low, SCAN-B TNBC
activated
suppressed
HALLMARK MYC TARGETS V1
HALLMARK MYC TARGETS V2
HALLMARK EPITHELIAL MESENCHYMAL TRANSITION
HALLMARK ANGIOGENESIS
HALLMARK E2F TARGETS
HALLMARK OXIDATIVE PHOSPHORYLATION
HALLMARK G2M CHECKPOINT
HALLMARK UNFOLDED PROTEIN RESPONSE
HALLMARK INTERFERON ALPHA RESPONSE
HALLMARK MTORC1 SIGNALING
HALLMARK DNA REPAIR
HALLMARK TGF BETA SIGNALING
HALLMARK HEDGEHOG SIGNALING
HALLMARK UV RESPONSE DN
HALLMARK APICAL JUNCTION
HALLMARK INTERFERON GAMMA RESPONSE
HALLMARK COAGULATION
HALLMARK MYOGENESIS
HALLMARK KRAS SIGNALING UP
HALLMARK BILE ACID METABOLISM
HALLMARK GLYCOLYSIS
HALLMARK HYPOXIA
HALLMARK XENOBIOTIC METABOLISM
HALLMARK SPERMATOGENESIS
HALLMARK MITOTIC SPINDLE
HALLMARK ADIPOGENESIS
Count
40
80
120
160
p.adjust
0.03
0.02
0.01
GeneRatio
C
Enriched GO terms in CAV1 High vs Low, SCAN-B TNBC
activated
suppressed
small-subunit processome
U2-type precatalytic spliceosome
organellar small ribosomal subunit
mitochondrial small ribosomal subunit
endonuclease complex
U4/U6 x U5 tri-snRNP complex
spliceosomal tri-snRNP complex
nuclear replication fork
protein complex involved in cell adhesion
collagen trimer
basement membrane
platelet alpha granule
collagen-containing extracellular matrix
cyclin-dependent protein kinase holoenzyme complex
caveola
platelet alpha granule lumen
plasma membrane raft
sarcolemma
endoplasmic reticulum lumen
kinesin complex
Count
50
100
150
200
p.adjust
0.00225
0.00200
0.00175
0.00150
0.00125
GeneRatio
D
Top DEG in CAV1 High vs Low, SCAN-B TNBC
CAV1HighLow
FABP4
ADH1B
CIDEA
AQP7
PLIN4
CIDEC
GPD1
ADIPOQ
PLIN1
ADH1C
VEGFD
OGN
HSPB6
CTSG
CCL14
C7
ABCA8
IGF2
COL17A1
MYH11
2
0
-2
CAV1HighLow
Low
High

S5 Fig

A

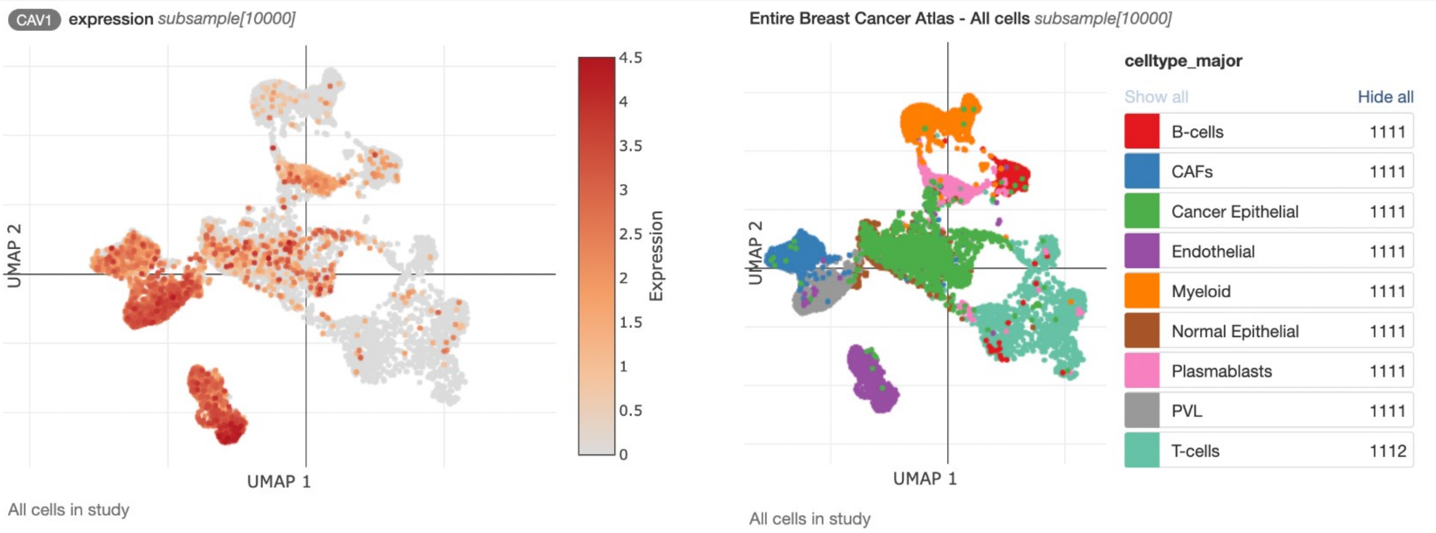

CAV1 expression subsample[10000]
UMAP 2
UMAP 1
Expression
4.5
4
3.5
3
2.5
2
1.5
1
0.5
0
All cells in study
Entire Breast Cancer Atlas - All cells subsample[10000]
celltype_major
Show all
Hide all
B-cells 1111
CAFs 1111
Cancer Epithelial 1111
Endothelial 1111
Myeloid 1111
Normal Epithelial 1111
Plasmablasts 1111
PVL 1111
T-cells 1112

B

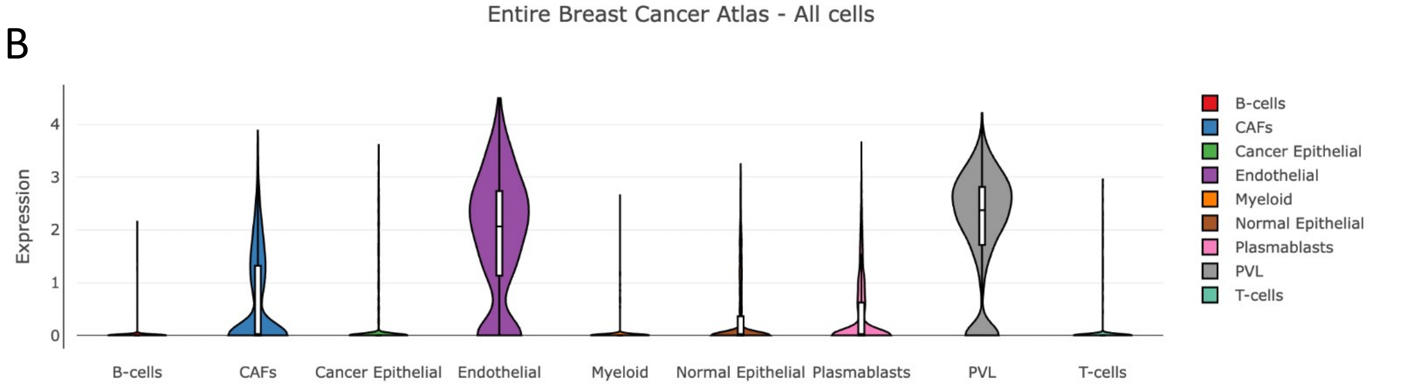

Entire Breast Cancer Atlas - All cells
Expression
B-cells
CAFs
Cancer Epithelial
Endothelial
Myeloid
Normal Epithelial
Plasmablasts
PVL
T-cells

C

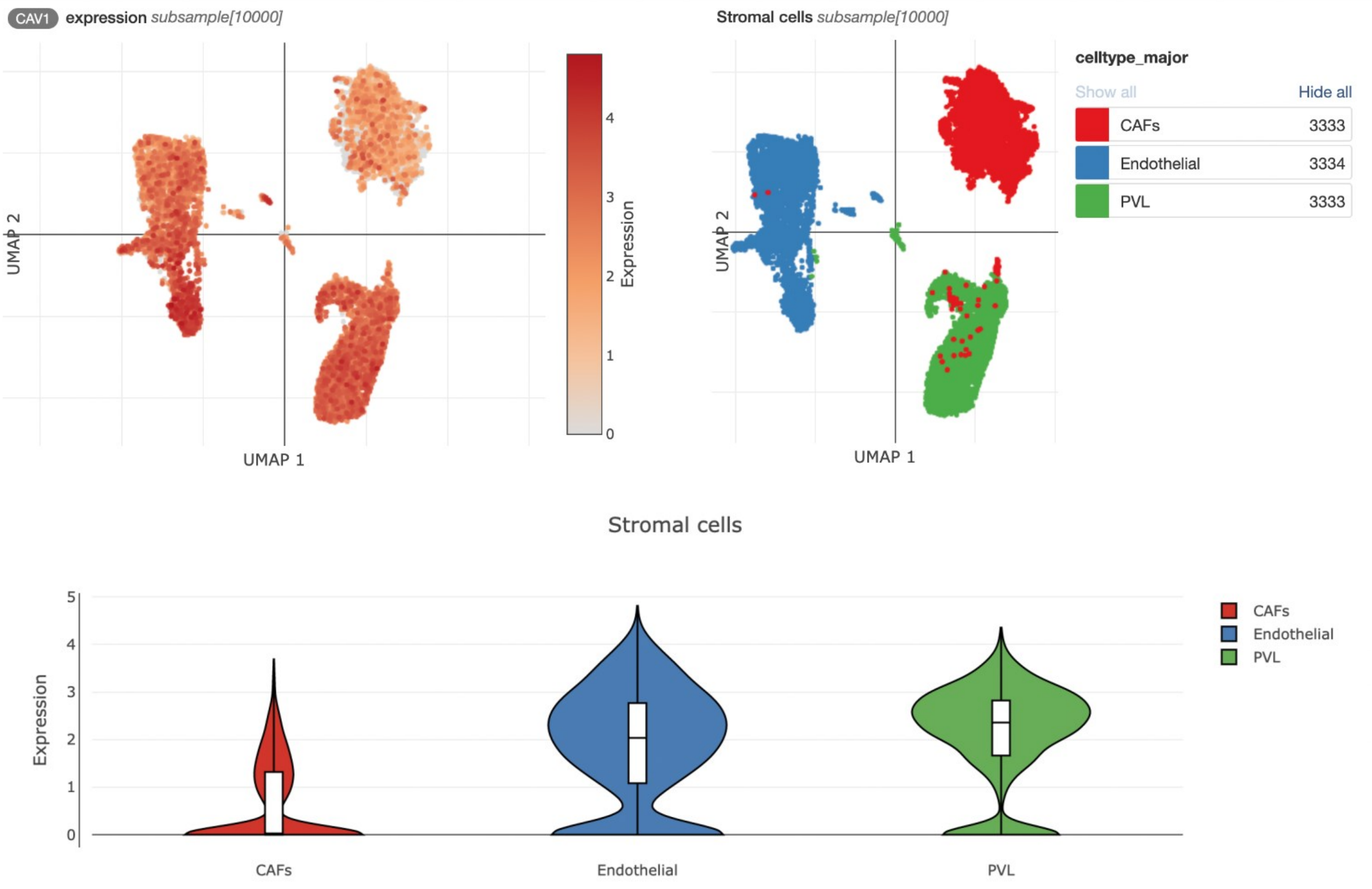

CAV1 expression subsample[10000]
UMAP 2
UMAP 1
Expression
Stromal cells subsample[10000]
celltype_major
Show all
Hide all
CAFs 3333
Endothelial 3334
PVL 3333
Stromal cells
CAFs
Endothelial
PVL

S6 Fig

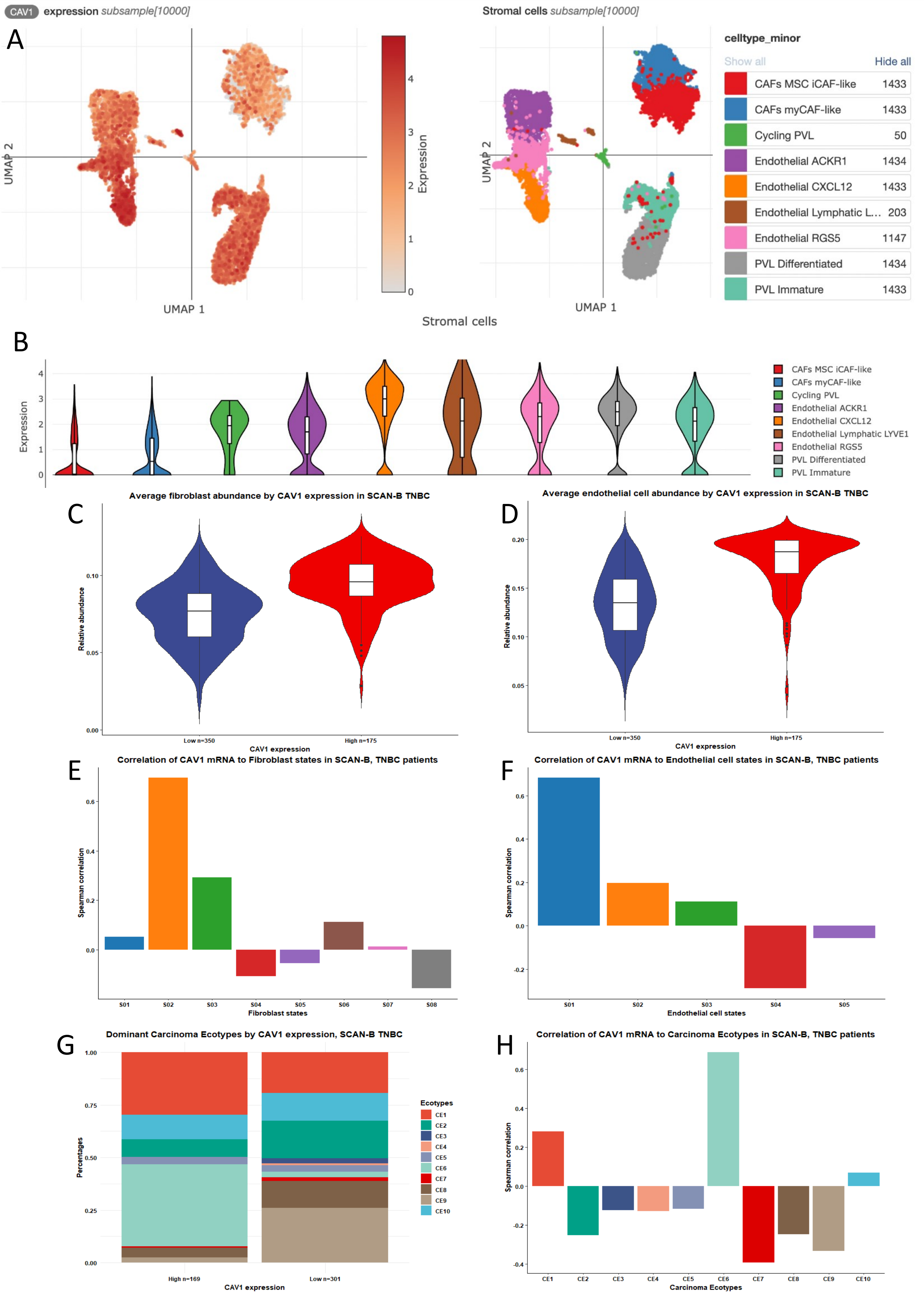

A
CAV1 expression subsample[10000]
UMAP 1
UMAP 2
Expression
0
1
2
3
4
Stromal cells subsample[10000]
celltype_minor
Show all
Hide all
CAFs MSC iCAF-like 1433
CAFs myCAF-like 1433
Cycling PVL 50
Endothelial ACKR1 1434
Endothelial CXCL12 1433
Endothelial Lymphatic L... 203
Endothelial RGS5 1147
PVL Differentiated 1434
PVL Immature 1433
Stromal cells
B
Expression
CAFs MSC iCAF-like
CAFs myCAF-like
Cycling PVL
Endothelial ACKR1
Endothelial CXCL12
Endothelial Lymphatic LYVE1
Endothelial RGS5
PVL Differentiated
PVL Immature
C
Average fibroblast abundance by CAV1 expression in SCAN-B TNBC
Relative abundance
Low n=350
High n=175
CAV1 expression
D
Average endothelial cell abundance by CAV1 expression in SCAN-B TNBC
Relative abundance
Low n=350
High n=175
CAV1 expression
E
Correlation of CAV1 mRNA to Fibroblast states in SCAN-B, TNBC patients
Spearman correlation
S01
S02
S03
S04
S05
S06
S07
S08
Fibroblast states
F
Correlation of CAV1 mRNA to Endothelial cell states in SCAN-B, TNBC patients
Spearman correlation
S01
S02
S03
S04
S05
Endothelial cell states
G
Dominant Carcinoma Ecotypes by CAV1 expression, SCAN-B TNBC
Percentages
Ecotypes
CE1
CE2
CE3
CE4
CE5
CE6
CE7
CE8
CE9
CE10
High n=169
Low n=301
CAV1 expression
H
Correlation of CAV1 mRNA to Carcinoma Ecotypes in SCAN-B, TNBC patients
Spearman correlation
Carcinoma Ecotypes

S7 Fig

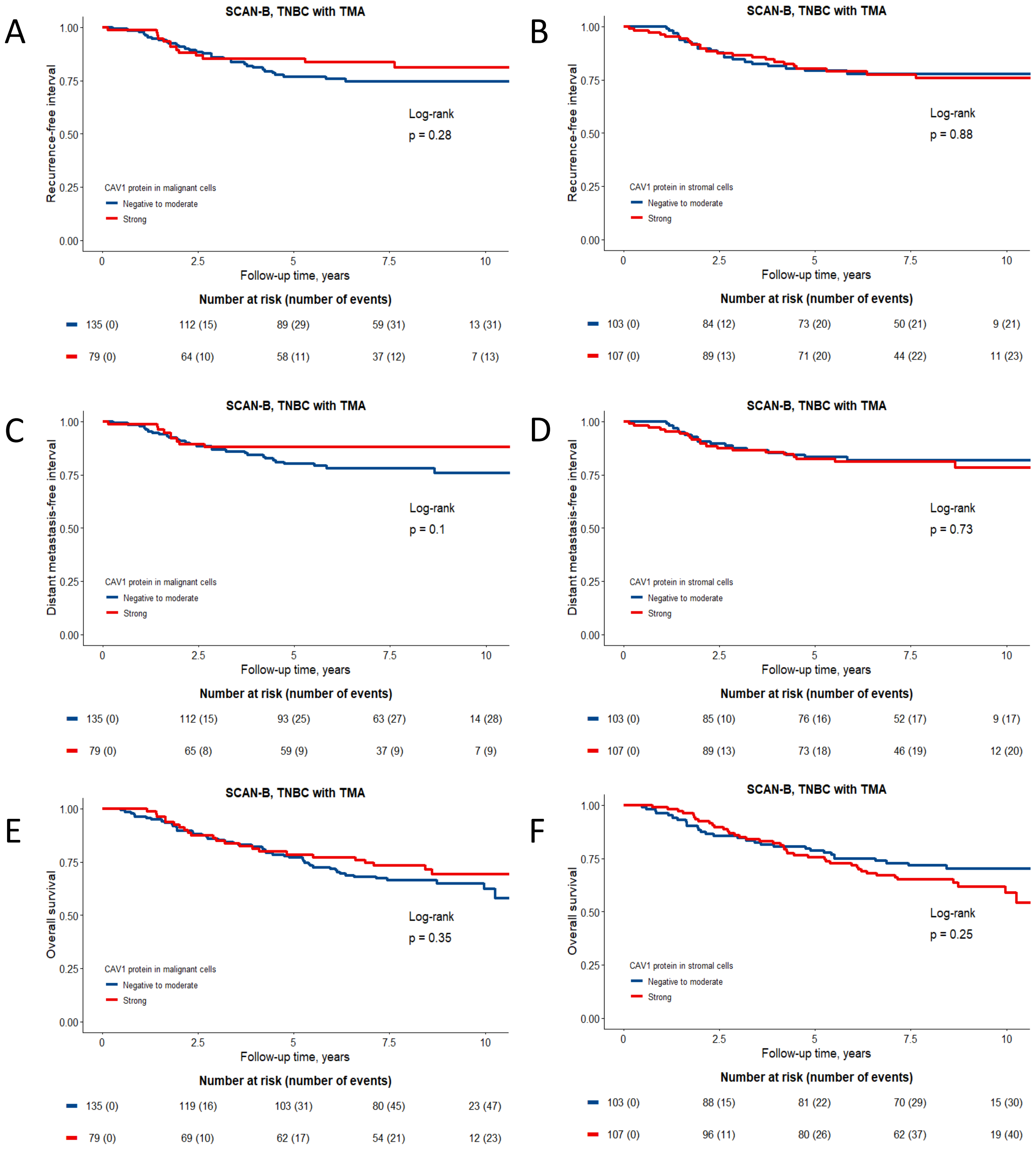
A
SCAN-B, TNBC with TMA
Recurrence-free interval
1.00
0.75
0.50
0.25
0.00
Log-rank
p = 0.28
CAV1 protein in malignant cells
Negative to moderate
Strong
0
2.5
5
7.5
10
Follow-up time, years
Number at risk (number of events)
135 (0) 112 (15) 89 (29) 59 (31) 13 (31)
79 (0) 64 (10) 58 (11) 37 (12) 7 (13)
B
SCAN-B, TNBC with TMA
Recurrence-free interval
Log-rank
p = 0.88
CAV1 protein in stromal cells
Negative to moderate
Strong
Follow-up time, years
Number at risk (number of events)
103 (0) 84 (12) 73 (20) 50 (21) 9 (21)
107 (0) 89 (13) 71 (20) 44 (22) 11 (23)
C
SCAN-B, TNBC with TMA
Distant metastasis-free interval
Log-rank
p = 0.1
CAV1 protein in malignant cells
Negative to moderate
Strong
Follow-up time, years
Number at risk (number of events)
135 (0) 112 (15) 93 (25) 63 (27) 14 (28)
79 (0) 65 (8) 59 (9) 37 (9) 7 (9)
D
SCAN-B, TNBC with TMA
Distant metastasis-free interval
Log-rank
p = 0.73
CAV1 protein in stromal cells
Negative to moderate
Strong
Follow-up time, years
Number at risk (number of events)
103 (0) 85 (10) 76 (16) 52 (17) 9 (17)
107 (0) 89 (13) 73 (18) 46 (19) 12 (20)
E
SCAN-B, TNBC with TMA
Overall survival
Log-rank
p = 0.35
CAV1 protein in malignant cells
Negative to moderate
Strong
Follow-up time, years
Number at risk (number of events)
135 (0) 119 (16) 103 (31) 80 (45) 23 (47)
79 (0) 69 (10) 62 (17) 54 (21) 12 (23)
F
SCAN-B, TNBC with TMA
Overall survival
Log-rank
p = 0.25
CAV1 protein in stromal cells
Negative to moderate
Strong
Follow-up time, years
Number at risk (number of events)
103 (0) 88 (15) 81 (22) 70 (29) 15 (30)
107 (0) 96 (11) 80 (26) 62 (37) 19 (40)
